# Multifaceted Sulfonamide-Derived Thiosemicarbazones: Combining Metal Chelation and Carbonic Anhydrases Inhibition in Anticancer Therapy

**DOI:** 10.3390/ijms26031225

**Published:** 2025-01-30

**Authors:** Mónica Martínez-Montiel, Giulia Arrighi, Paloma Begines, Aday González-Bakker, Adrián Puerta, Miguel X. Fernandes, Penélope Merino-Montiel, Sara Montiel-Smith, Alessio Nocentini, Claudiu T. Supuran, José M. Padrón, José G. Fernández-Bolaños, Óscar López

**Affiliations:** 1 Departamento de Química Orgánica, Facultad de Química, Universidad de Sevilla, Apartado 1203, E-41071 Seville, Spain; monica.martinezmon@alumno.buap.mx (M.M.-M.); giulia.arrighi1@stud.unifi.it (G.A.); pbegines@us.es (P.B.); bolanos@us.es (J.G.F.-B.); 2 Facultad de Ciencias Químicas, Ciudad Universitaria, Benemérita Universidad Autónoma de Puebla, Puebla 72570, PUE, Mexico; penelope.merino@correo.buap.mx (P.M.-M.); maria.montiel@correo.buap.mx (S.M.-S.); 3 NEUROFARBA Department, Sezione di Scienze Farmaceutiche e Nutraceutiche, University of Florence, 50019 Florence, Italy; alessio.nocentini@unifi.it (A.N.); claudiu.supuran@unifi.it (C.T.S.); 4 BioLab, Instituto Universitario de Bio-Orgánica “Antonio González” (IUBO-AG), Universidad de La Laguna, c/Astrofísico Francisco Sánchez 2, E-38206 La Laguna, Spain; agonzaba@ull.es (A.G.-B.); apuertaa@ull.es (A.P.); mfernand@ull.edu.es (M.X.F.); jmpadron@ull.es (J.M.P.)

**Keywords:** carbonic anhydrases, sulfonamides, thiosemicarbazones, docking simulations, metal chelation, antiproliferative activity

## Abstract

The selective inhibition of key enzymes, such as carbonic anhydrases (CAs IX and XII), which are overexpressed in cancer tissues, has emerged as a promising strategy in cancer research. However, a multitarget approach is often preferred to achieve enhanced therapeutic outcomes. In this study, aryl sulfonamides were conjugated with a thiosemicarbazone moiety to enable dual functionality: the inhibition of CAs and the chelation of metal cations. Several structural factors were systematically modified, including the position of the sulfonamido group, the length of the linker, the nature of the aromatic residue, and the type of substituents. Tumor-associated CAs IX and XII inhibition was evaluated using the stopped-flow CO_2_ hydrase assay, and the inhibition constants (*K_i_*) were determined. The most promising compounds were further analyzed through molecular docking simulations. Metal chelation capabilities were evaluated using UV–Vis spectroscopy, while antiproliferative activities were measured using the sulforhodamine B (SBR) assay. Additionally, holotomographic 3D microscopy was employed to investigate the mechanisms of cell death. Sulfonamido-derived Schiff bases were synthesized through a three-step procedure that did not require column chromatography purification: (1) isothiocyanation of amino-sulfonamides, (2) nucleophilic addition of hydrazine, and (3) acid-promoted condensation with different aldehydes (benzaldehydes or pyridine-2-carboxaldehyde). The synthesized compounds exhibited inhibition of CAs in the low nanomolar to submicromolar range, with selectivity largely influenced by structural features. Notably, the *m*-sulfonamide derivative **5b**, bearing a pyridin-2-yl residue, demonstrated potent and selective inhibition of CA IX (*K*_i_ = 4.9 nM) and XII (*K*_i_ = 5.6 nM). Additionally, it efficiently chelated Fe^2+^, Fe^3+^, and Cu^2+^ and showed promising antiproliferative activity (GI_50_ 4.5–10 µM). Mechanistic studies revealed that apoptosis was involved in its mode of action. Therefore, the synergistic integration of sulfonamides and thiosemicarbazones represents an effective strategy for the development of multimodal anticancer agents.

## 1. Introduction

Cancer is the second leading cause of mortality worldwide [1], representing one of the most significant challenges in biomedical research [2]. It is among the most complex and devastating diseases of our time [3]. Despite tremendous advances in early detection [4], surgery [5], radiotherapy [6], immunotherapy [7], nanotechnology [8] and other therapeutic approaches, chemotherapy continues to be a cornerstone treatment for many types of cancer [9]. However, the intricate complexity of cancer, combined with its multifactorial etiology, makes the traditional one-drug, one-target paradigm [10] insufficient. Consequently, a multitarget strategy [11] has become essential for combating tumor progression and therapeutic resistance [12].

In this context, our study aimed to develop multimodal therapeutic agents for cancer treatment. Specifically, we sought to design compounds that integrate a pharmacophore targeting enzymes overexpressed in tumors with a metal chelator to address the high levels of certain metals in tumor tissues. Our primary focus was on carbonic anhydrases (CAs), a family of ubiquitous Zn(II)-dependent metalloenzymes. These enzymes catalyze the reversible hydration of CO_2_ to produce hydrogen carbonate and a proton [13]. The Zn(II) cation acts as a Lewis acid, lowering the p*K*_a_ of the coordinated H_2_O molecule, thereby facilitating its deprotonation under physiological conditions [14]. CAs are found across almost all domains of life and are classified into eight different genetic families. The ones found in humans (α-CAs) are in turn divided into 15 different isoforms, which vary in tissue distribution and catalytic activity [15]. The selective inhibition of the α-CAs IX and XII isoforms has emerged as a promising target in anticancer research [16], due to their role in acidifying the hypoxic tumor microenvironment, which promotes tumor progression and metastasis [17]. Alkyl and aryl sulfonamides constitute the largest family of CA inhibitors [18], which act by binding Zn(II) and thus blocking the enzyme activity. Interestingly, this pharmacophore has also been found to inhibit tyrosine kinases and aromatases [19], both of which are overexpressed in certain tumors. A notable example is compound SLC-0111, a ureido-containing aryl sulfonamide and a potent CA IX inhibitor currently undergoing clinical trials for advanced solid tumors. SLC-0111 has demonstrated potential as a sensitizer for head and neck squamous cell carcinoma (HNSCC) in combination with cisplatin [20] and has demonstrated the capacity of reducing hepatoblastoma cell viability and migration [21].

In addition to targeting CAs, we explored the incorporation of a metal-chelating framework linked to the sulfonamido motif. To the best of our knowledge, no prior studies have described anticancer agents designed to simultaneously target CAs and the disruption of metal homeostasis. Metals play crucial roles in healthy cells [22], contributing to signalling pathways [23], enzymatic activity, and the structural integrity of cell membranes and the genome [24]. However, disruptions in metal homeostasis—characterized by either an excess or deficiency—can profoundly affect cellular physiology. Elevated levels of metal cations such as Fe, Cu, and Zn have been linked to oncogenesis and metastasis [24,25]. For instance, high levels of Fe can exacerbate oxidative stress by interacting with H_2_O_2_, causing severe damage to cellular membranes and organelles [26].

Although further research is needed, metal chelators have shown promising results in preclinical anticancer studies [27,28]. Schiff bases and their derivatives, particularly thiosemicarbazones (the scaffold used in this study), have attracted significant attention as metal-binding ligands in anticancer research [29,30,31], with several compounds entering clinical trials [32]. Metal complexes derived from thiosemicarbazones have been extensively characterized using diverse analytical techniques, including IR and NMR spectroscopy, conductivity measurements, thermogravimetric analysis, and density functional theory (DFT) calculations [33,34].

## 2. Results and Discussion

### 2.1. Drug Design and Chemistry

**Figure 1:**
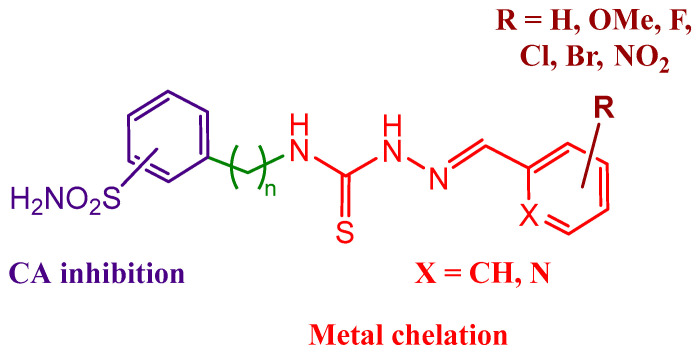
Design of multifaceted sulfonamide-derived thiosemicarbazones.

The primary objective of this study was the design, synthesis, and evaluation of multitarget sulfonamide–thiosemicarbazone hybrids (Figure 1) with potential anticancer activity, specifically targeting tumor-related CAs and hazardous high levels of metals. This approach represents a promising strategy for developing multitarget drug candidates with potential application in cancer chemotherapy. It has been suggested that the antineoplastic properties of thiosemicarbazones are enhanced when they exert effective metal-chelating capabilities [35]. Schiff bases and related compounds, particularly thiosemicarbazones, have been reported to exhibit anticancer properties through various mechanisms [36], including interactions with DNA [37], inhibition of topoisomerases [38], and mitigation of multidrug resistance (MDR) [32]. Among these mechanisms, their capacity to complex iron is widely recognized as the primary anticancer mechanism [39], with Fe-dependent ribonuclease reductase (RNR) identified as a key intracellular target. Recent studies on nanomolar anticancer thiosemicarbazones have also highlighted their ability to complex Cu(II) ions, with human serum albumin identified as a potential metal source [40]. Consequently, thiosemicarbazones are now regarded as more than just simple chelators; they are classified as metal-interacting drugs with multimodal anticancer activity [41]. A notable example of this pharmacophore is Triapine^®^ (3-aminopyridine-2-carboxaldehyde thiosemicarbazone), which has undergone numerous clinical trials [42]. In this study, the two key structural moieties investigated were the aryl sulfonamide and the thiosemicarbazone groups. To further enhance biological activity, structural modifications were systematically introduced, including varying the position of the sulfonamido motif, employing different spacers to link the sulfonamide and thiosemicarbazone moieties, incorporating aryl and heteroaryl residues (e.g., pyridine-2-yl) into the imino scaffold, and modifying substituents on the aryl ring. These structural variations provided a robust framework for establishing structure–activity relationships (SARs), facilitating the rational design of more potent and selective anticancer agents.

**Scheme 1:**
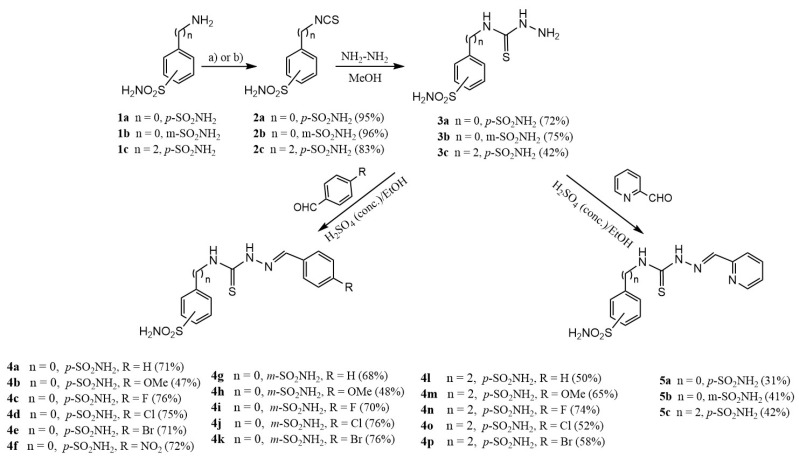
Preparation of sulfonamide-derived thiosemicarbazones **4** and **5**. (a) CSCl_2_, aq. HCl; (b) DCC, CS_2_, Py.

The synthetic pathway is outlined in Scheme 1. Commercially available aryl amino-sulfonamides **1a–c** were transformed into their corresponding isothiocyanates using two different procedures: treatment with thiophosgene under acidic conditions [43] for derivatives **1a** and **1b**, or reaction with dicyclohexyl carbodiimide (DCC) and CS_2_ in Py [44] for compound **1c**. The resulting heterocumulenes (compounds **2a–c**, obtained in good to excellent yields) were subsequently treated with hydrazine hydrate to furnish the corresponding thiosemicarbazides **3a–c**, with yields ranging from 42 to 75%. Final condensation under acidic conditions with benzaldehydes or pyridine-2-carboxaldehyde yielded sulfonamide-derived thiosemicarbazones **4a–p** (47–76%) or **5a–c** (31–42%), respectively. The incorporation of a pyridine-2-yl residue in derivatives **5a–c** is expected to provide a tridentate ligand, likely capable of complexing relevant cations more efficiently. This property may facilitate the sequestration and removal of metal ions from tumor tissues. The synthetic procedure is efficient and straightforward, with all intermediates and final compounds being crystalline and easily purified by filtration. This eliminates the need for time-consuming column chromatography purifications, making the process highly practical for further development.

**Figure 2:**
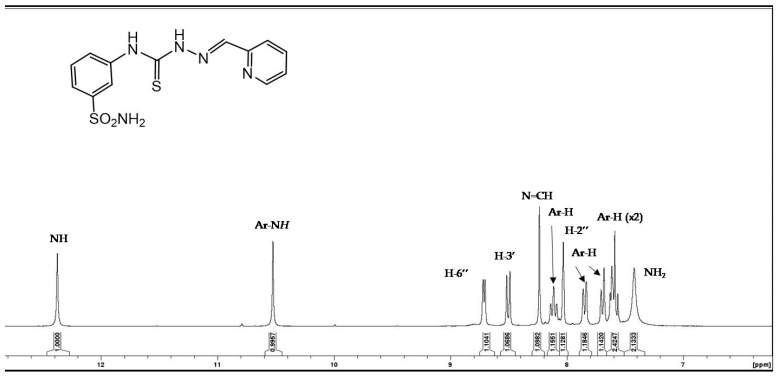
^1^H-NMR spectrum of **5b** (300 MHz, DMSO-*d*_6_).

**Figure 3:**
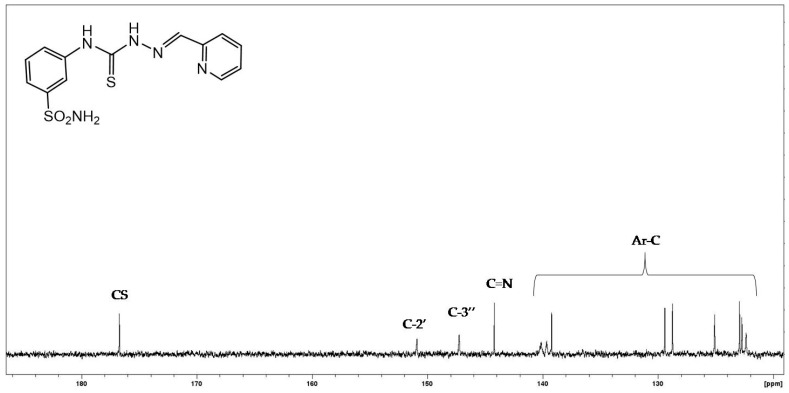
^13^C-NMR spectrum of **5b** (75.5 MHz, DMSO-*d*_6_).

As a representative example, Figure 2 and Figure 3 depict the ^1^H- and ^13^C-NMR spectra of the pyridine-containing thiosemicarbazone **5b**. In the ^1^H-RNM spectrum, the most notable signals are observed at 12.34 ppm (Ar-NH), 10.51 ppm (NH), and 8.21 ppm (azomethyne proton). Similarly, in the ^13^C-NMR spectrum, the resonances at 176.7 ppm (thione moiety) and 144.2 ppm (imine moiety) provide further confirmation of the proposed structure.

### 2.2. Biological Assessments

#### 2.2.1. CA Inhibition

Thiosemicarbazones **4a–p** and **5a–c** were evaluated as potential inhibitors of tumor-associated CAs IX and XII. For comparison, their synthetic precursors, thiosemicarbazides **3a–c**, as well as acetazolamide (AAZ), as a standard positive control, were included in the study. To assess selectivity, the inhibition of cytosolic CAs I and II was also measured. The inhibition constants (*K*_i_, nM), determined using the stopped-flow CO_2_ hydrase assay, along with the selectivity indexes, are depicted in Table 1. The following SARs were identified:

Inhibition of CAs IX and XII: all Schiff bases exhibited varying degrees of inhibition of transmembrane CAs IX and XII, with *K*_i_ values ranging from low nanomolar to submicromolar (2.3–499 nM), depending on the aryl ring substituents, the position of the sulfonamido moiety, and the presence or absence of an ethylene linker.

Preference for CA XII: across all derivatives, including thiosemicarbazide precursors **3**, stronger inhibition was consistently observed for CA XII compared with CA IX. This trend is particularly noteworthy, as numerous CA XII inhibitors are also known to inhibit glycoprotein-P (Pg-p), potentially reducing chemoresistance caused by xenobiotic efflux via the Pg-p pump [45]. An exception to this trend was observed for the pyridine-derived thiosemicarbazone **5b**, which displayed comparable potency against both isoforms (*K*_i_ = 4.9 and 5.6 nM, respectively).

*m*- and *p*-Substituted sulfonamido derivatives: among compounds bearing the sulfonamido motif at the *para* position, the highest activity against CA XII was observed for unsubstituted aromatic rings (**4a**, *K*_i_ = 8.38 nM; **4l**, *K*_i_ = 9.12 nM). The introduction of an ethylene linker (**4l–p** vs. **4a–f**) enhanced selectivity for CA XII, primarily by reducing activity against CA I, with *K*_i_ values in the micromolar range for derivatives **4m–p**. Among the *meta* regioisomers, derivative **4h**, bearing a *p*-methoxy substituent, exhibited the highest CA XII activity (*K*_i_ = 2.3 nM).

Pyridine-containing derivatives: thiosemicarbazones containing a pyridine fragment and lacking the ethylene linker (**5a,b**) displayed strong inhibition of CA XII (*K*_i_ = 4.9 and 5.6 nM, respectively), with selectivity comparable to or exceeding that of the reference drug AAZ. Additionally, compound **5b** exhibited potent inhibition of CA II (*K*_i_ = 2.5 nM). Notably, CAs IV, XII, and, particularly, II, are validated targets for glaucoma treatment due to their role in mitigating ocular hypertension [46]. Incorporation of the ethylene linker (**5c**) preserved strong inhibition of CA XII (*K*_i_ = 9.3 nM) but significantly decreased CA I/XII selectivity.

Selectivity: the *meta* placement of the sulfonamido moiety on the aromatic ring generally reduced activity against CA I, thereby improving selectivity. This is a critical factor for minimizing off-target effects. Remarkably, the strongest CA XII inhibitor, derivative **4h** (*K*_i_ = 2.3 nM), exhibited the highest CA I/XII selectivity index (S.I. = 325), significantly outperforming AAZ (*K*_i_ = 5.2 nM for CA XII; S.I. = 43.9). Another noteworthy compound is the *m*-sulfonamide **5b**, which bears a pyridine-2-yl scaffold (S.I. = 109.8 and 96.1 for CA I/IX and CA I/XII, respectively).

These findings align with previously reported data, in which many aryl sulfonamides demonstrated significant potency as inhibitors of CA IX and XII isoforms, with *K*_i_ values in the low nanomolar range. However, their selectivity against off-target isoforms, such as CA I, is usually lower compared with coumarins [47], another important class of CA inhibitors.

#### 2.2.2. Docking Simulations

The interaction profiles of the selected thiosemicarbazone–sulfonamide hybrids with CA IX and CA XII enzymes were analyzed using ligand–protein docking simulations. The simulations took into account the water molecules surrounding the Zn(II) ion, in line with the widely reported interaction mechanisms for sulfonamide-based CA inhibitors. Additionally, based on extensive literature evidence, the sulfonamido moiety of the hybrids was deprotonated for the simulations. For this study, derivatives **4a** and **5b** were considered as representative compounds. The binding energies from the docking simulations against CA IX are shown in Table 2.

**Figure 4:**
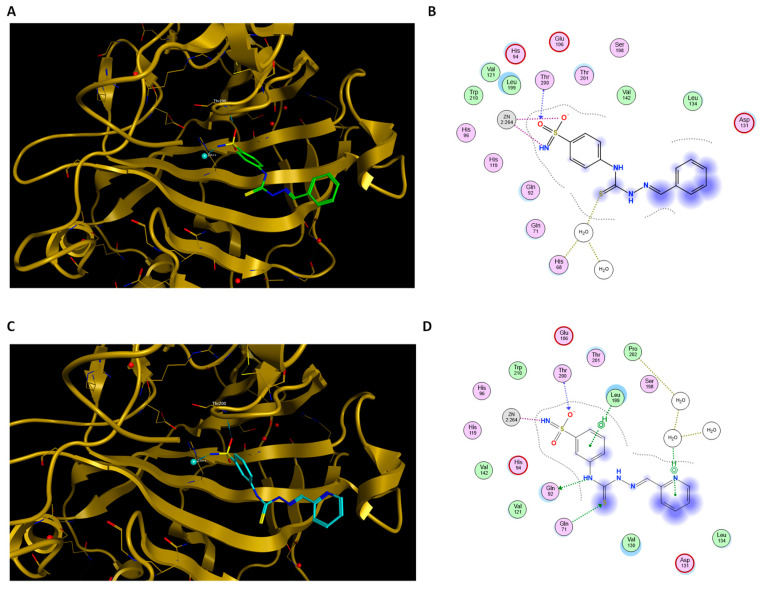
(**A**,**C**): docking representation of CA IX (gold)–**4a** (green) and **5b** (light blue) binding complex. (**B**,**D**): two-dimensional scheme of the protein–ligand interactions.

Docking simulations predict that both **4a** and **5b** interact with the Zn(II) ion through the deprotonated form of the sulfonamido motif and with the gatekeeper residue Thr200. Additionally, the partially negative oxygen atom of **4a** also interacts with the Zn(II) ion, while the sulfur atom of the thiosemicarbazone moiety is involved in an H-bond between water and His68. In contrast, for **5b**, the same sulfur atom interacts with Gln71 as an electron acceptor, whilst the Gln92 is the acceptor from an interaction with the NH group of the thiosemicarbazone. Moreover, an H-π stacking interaction occurs between the aromatic moiety of the benzenesulfonamide and Leu199. The heteroatom of the pyridine ring also participates in cooperative H-bonding with water molecules and Pro202 (Figure 4).

**Figure 5:**
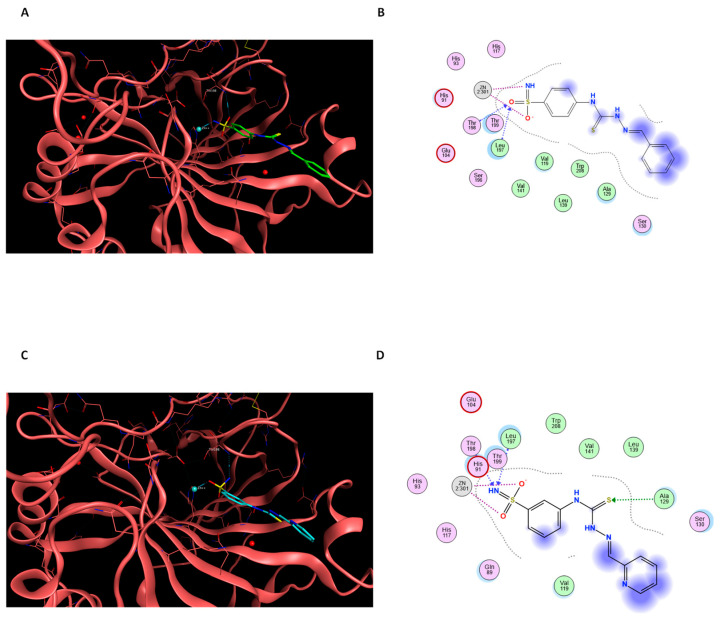
(**A**,**C**): docking representation of CAXII (pink)—**4a** (green) and **5b** (light blue) binding complex. (**B**,**D**): two-dimensional scheme of the protein–ligand interactions.

Similar to the findings observed for CA IX, the deprotonated NH of the sulfonamido moiety in **4a** and **5a** interacts with the Zn(II) ion and simultaneously with Leu197 and the gatekeeper residue Thr198 in CA XII. Additionally, compound **5b** exhibits a direct binding with Ala 129 (Figure 5). The binding energies for the interaction with CA XII are depicted in Table 3.

#### 2.2.3. Metal Complexation Assays

**Figure 6:**
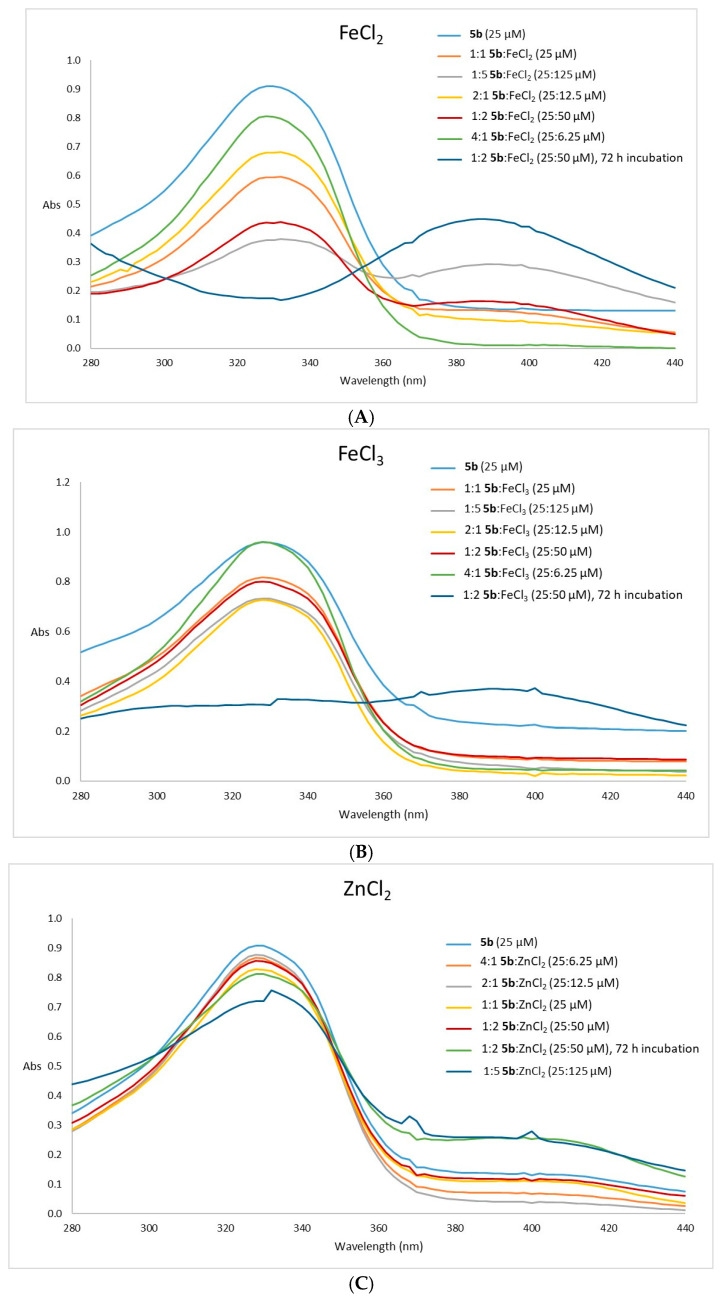

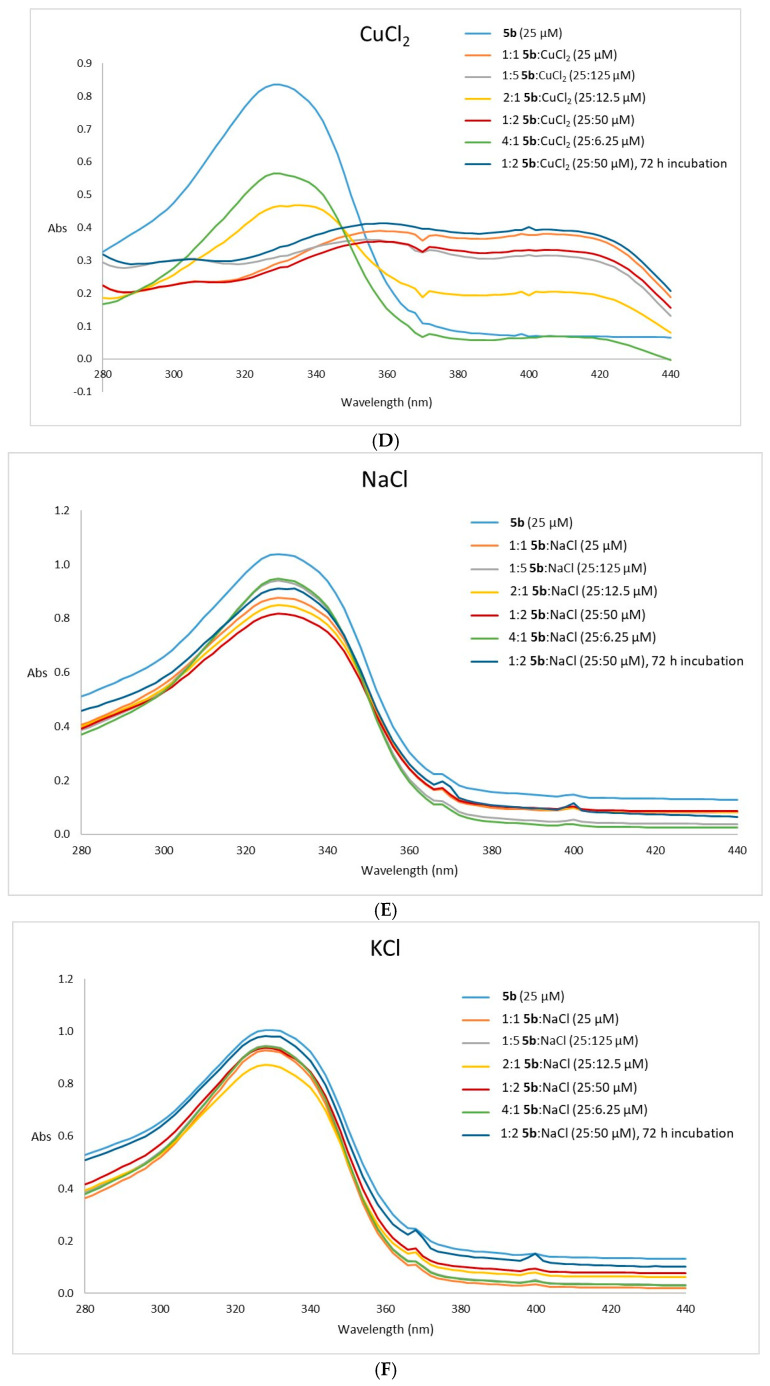
UV—Vis spectra of **5b**—metal chlorides at different ratio. (**A**) FeCl_2_; (**B**) FeCl_3_; (**C**) ZnCl_2_; (**D**) CuCl_2_; (**E**) NaCl; (**F**) KCl.

The ability of **5b**, as a model compound, to complex metal cations associated with tumorigenesis (Na^+^, K^+^, Fe^2+^, Fe^3+^, Zn^2+^, and Cu^2+^) was analyzed using UV–Vis spectroscopy. To investigate this, UV–Vis spectra of solutions with varying ratios of **5b** to metal chlorides (1:0, 4:1, 2:1, 1:1, 1:2, 1:5) were recorded. Additionally, a 1:2 ligand-to-metal spectrum was obtained after 72 h of incubation to account for potential slow complexation. Titration experiments with NaCl and KCl showed no significant spectral changes with increasing amounts of the metals (Figure 6E,F, respectively), indicating that **5b** does not chelate these monovalent cations. In contrast, the spectra for divalent and trivalent cations revealed a decrease in absorbance at λ_max_, accompanied by a significant bathochromic shift. These changes suggest that **5b** chelates Fe^2+^, Fe^3+^, Zn^2+^, and Cu^2+^ ions. Among these, the most pronounced effect was observed with CuCl_2_ (Figure 6B). The addition of stoichiometric or excess amounts of CuCl_2_ resulted in a marked increase in λ_max_ and significant flattening of the spectrum. FeCl_2_ and FeCl_3_ also induced a decrease in absorbance at λ_max_ with noticeable spectrum flattening and a bathochromic shift after incubation, likely reflecting the formation of a slow ligand–metal complex (Figure 6A,B). For ZnCl_2_, only a weak effect was observed after incubation, and no significant changes were detected without incubation, regardless of the ligand-to-Zn^2+^ ratio (Figure 6C). In summary, these findings demonstrate that thiosemicarbazone **5b** is an efficient ligand for chelating hazardous levels of Cu^2+^ and, to a lesser extent, Fe^2+^ and Fe^3+^. Its affinity for Zn^2+^ is notably weaker, suggesting limited efficacy in binding this metal under the tested conditions.

**Figure 7:**
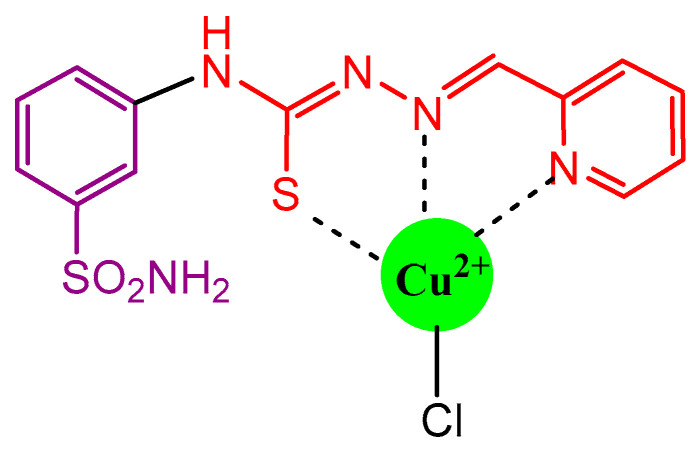
Postulated structure of metal complexes involving **5b** as a tridentate ligand.

Based on previous reports [48,49] describing metal complexes of pyridine-2-carboxaldehyde thiosemicarbazones, we postulate that compound **5b** acts as an S,N,N_py_ tridentate ligand in its complexation with the di- and trivalent metals tested (Figure 7). The capacity of thiosemicarbazone ligands to exacerbate ROS levels upon complexation [50] or to block Fe-containing enzymes, such as RNR [39], cannot be discarded.

#### 2.2.4. Antiproliferative Assay

The potential antiproliferative activity of sulfonamides synthesized in this study was evaluated in vitro using the SBR assay, following the US NCI protocol. A panel of six tumor cell lines was employed: A549 (non-small cell lung), HBL-100 (breast), HeLa (cervix), SW1573 (non-small cell lung), T-47D (breast), and WiDr (colon). The latter two represent multidrug-resistant cell lines. The results are summarized in Table 4, and the following SARs were identified:

Thiosemicarbazide precursors (compounds **3**) were completely inactive across all tested cell lines.

Increasing the distance between the benzenesulfonamide scaffold and the thiosemicarbazone functionality (n = 0, 2) caused a significant reduction in activity for both non-substituted derivatives and those containing a pyridine moiety. The observed activities ranged from good (low micromolar range) for the shortest distance to almost inactive for the longest distance (**4a** vs. **4l**; **5a** vs. **5c**). The elimination of the ethylene linker in pyridine-based thiosemicarbazones (compounds **5a** and **5b**) compared with **5c** led to a significant enhancement in activity, achieving GI_50_ values < 10 µM in five out of six cell lines.

The position of the sulfonamido motif on the aromatic ring emerged as a critical structural determinant in thiosemicarbazones lacking the pyridin-2-yl residue (compounds **4**). Generally, *p*-sulfonamido derivatives exhibited superior activity compared with their corresponding *m*-regioisomers. This effect was particularly evident in compound **4a** (R = H), which demonstrated potent antiproliferative activity with GI_50_ values ranging from 2.3 to 5.1 µM across all tested cell lines. In contrast, the corresponding *m*-isomer, compound **4g**, exhibited only modest activity (GI_50_ = 51 to >100 µM).

Introducing a polar, strong electron-donating group (OMe) on the phenyl ring of the imine fragment had a detrimental effect on activity. This modification resulted in either inactive derivatives or compounds with only moderate activity (compounds **4b,h,m**).

Overall, the most potent Schiff bases in terms of antiproliferative activity were the non-substituted derivative **4a** and the pyridine-derived compounds with the shortest linkers (compounds **5a,b**). Notably, these compounds displayed enhanced activity against multidrug-resistant cell lines (T-47D and WiDr) compared with the reference drugs 5-fluorouracil and cisplatin, demonstrating a 7- to 13-fold increase in potency. The observation that derivatives with weak antiproliferative properties, such as precursors **3** or thiosemicarbazones **4b,d,f,h,i,m,n** exhibit good inhibition of CA IX and XII suggests that these metalloenzymes are not the sole targets in their mode of action. Thiosemicarbazones **5a** and **5b**, which feature a pyridine-2-yl moiety, were found to be effective antiproliferative agents. However, their activity was lower compared with other pyridine-2-carboxyaldehyde thiosemicarbazones reported in the literature that lack the sulfonamido motif [51,52]. This suggests that the incorporation of a polar group with a strong electron-withdrawing effect has a detrimental impact on antiproliferative properties. Nevertheless, the lead compounds **4a** and **5b**, which still retain good antiproliferative activity, exhibit a dual capacity to inhibit tumor-associated carbonic anhydrases CA IX and XII and to reduce hazardous levels of di- and trivalent cations. This multifunctional profile positions them as promising candidates for addressing the multifactorial nature of cancer.

#### 2.2.5. Label-Free Continuous Live Cell Imaging

**Figure 8:**
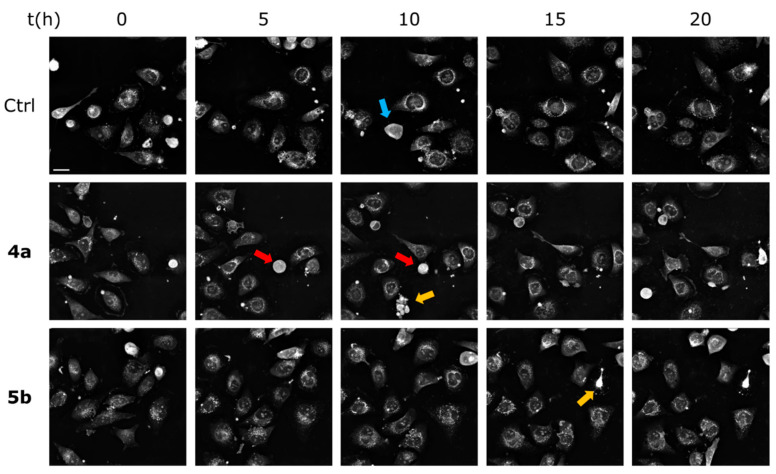
SW1573 cells were subjected to treatment with **4a** and **5b** for 20 h. Representative images for each treatment at different time intervals are shown. Yellow arrows indicate the presence of apoptotic cells. Red arrows denote cells unable to undergo mitosis. Blue arrows show cells undergoing mitosis. Scale bar: 30 µm.

Live imaging allows researchers to observe progressive cytostatic effects [53] or cell death of treated cells, enabling the identification of several apoptotic hallmarks [54,55]. In order to characterize the phenotypic effects of the compounds **4a** and **5b** on cancer cells, the evolution of the culture was monitored every 3 min using label-free holotomographic 3D microscopy. SW1573 cells were exposed to **4a** and **5b** at a concentration of 20 μM for 20 h (Supplementary Material, videos S1–S3). Untreated SW1573 cells (control) moved freely and divided throughout the experimental time frame. In contrast, in **4a** and **5b** treated samples, cell division was retarded and apoptotic features were observed in some cells (Figure 8).

## 3. Materials and Methods

### 3.1. Chemistry

#### 3.1.1. General Methods

TLCs were performed using aluminum-coated sheets (Merck 60 F_254_), 0.25 mm width. Each eluant is indicated in the experimental procedures. Spots were visualized by UV light (λ = 254 nm) and by charring with 10% ethanolic vanillin containing 1% H_2_SO_4_ or with 5% ethanolic phosphomolybdic acid. Column chromatography purifications were performed using silica gel stationary phase (Merck 60, particle size 40–63 μm), eluting by gravity or with mild pressure. Eluants are indicated in each case. NMR spectra were registered in the Centro de Investigación, Tecnología e Innovación de la Universidad de Sevilla (CITIUS), using Bruker Avance III 300 spectrometer (300.1 MHz for ^1^H, 75.5 MHz for ^13^C) and DMSO-*d*_6_ as solvent (Darmstadt, Germany). Assignments were confirmed by using 2D homo- and heteronuclear experiments (COSY, HSQC). Chemical shifts (δ) are expressed in ppm, and coupling constants (*J*) are expressed in Hz. Residual signals from the solvent are used as internal references [56] for the calibration (2.50 ppm for ^1^H and 39.5 ppm for ^13^C). Mass spectra were registered using a Qexactive spectrometer using Electrospray Ionization (ESI). Sample injection (MeOH as solvent) was performed using UHPLC without column (50 μL and acquisition for 3 min at 0.200 mL/min). Acquisition is carried out with full scan at 60000 resolution. Capillary temperature was 350 °C and the source voltage, 3.5 kV. Spectra were registered under positive mode and calibrated using the Pierce™ LTQ Velos ESI Positive Ion Calibration Solution (ThermoFisher Scientific, Waltham, MA, USA).

#### 3.1.2. General Procedure for the Preparation of Isothiocyanates **2a,b** [43]

To a solution of the corresponding aminobenzenesulfonamide (**1a,b**) (500 mg, 3.0 mmol, 1.0 equiv.) in H_2_O (50 mL) and 37% aq. HCl (12 mL) was added thiophosgene (0.20 mL, 3.0 mmol, 1.0 equiv.), and the solution was stirred until the reddish color of the solution disappeared. A precipitate was formed, and it was filtered and washed with cold water. The filtrate was recrystallized from a H_2_O–acetone mixture.

4-Sulfonamidophenyl isothiocyanate (**2a**). Yield: 585 mg (91%) [44].

3-Sulfonamidophenyl isothiocyanate (**2b**). Yield: 617 mg (96%) [44].

#### 3.1.3. 4-(2′-Isothiocyanatoethyl)Benzenesulfonamide (2c) [44]

To a solution of 4-(2′aminoehtyl)benzenesulfonamide (**1c**) (500 mg, 2.5 mmol) and DCC (500 mg, 2.4 mmol) in Py (1 mL) kept at 0–10 °C was added CS_2_ (2 mL, 33 mmol) in Py (1 mL). The corresponding solution was stirred at rt for 12 h. After that, the solvent was eliminated in vacuo, and the residue was crystallized from acetone. Yield: 415 mg (83%).

#### 3.1.4. General Procedure for the Preparation of Thiosemicarbazides **3a–c**

To a solution of the corresponding isothiocyanate **2a–c** (1.0 equiv.) in MeOH (2 mL) was dropwise added a solution of monohydrated hydrazine (1.2 equiv.) in MeOH (2 mL). After the addition, the mixture was stirred at rt for 4 h, and a precipitate was formed, which was filtered and washed with cold MeOH. The products were used without further purification for the next step.

4-*N*-(4′-Sulfonamidophenyl)-3-thiosemicarbazide (**3a**). Isothiocyanate **2a** (100 mg, 0.46 mmol, 1.0 equiv.) and hydrazine (26 µL, 0.55 mmol, 1.2 equiv.) were used. Compound **3a** was obtained as a white solid. Yield: 83 mg (72%) [57].

4-*N*-(3′-Sulfonamidophenyl)-3-thiosemicarbazide (**3b**). Isothiocyanate **2b** (100 mg, 0.46 mmol, 1.0 equiv.) and hydrazine (26 µL, 0.55 mmol, 1.2 equiv.) were used. Compound **3b** was obtained as a white solid. Yield: 87 mg (75%) [58].

4-*N*-[2′-(4″-Sulfonamido)phenyl]ethyl-3-thiosemicarbazide (**3c**). Isothiocyanate **2c** (100 mg, 0.41 mmol, 1.0 equiv.) and hydrazine (24 µL, 0.49 mmol, 1.2 equiv.) were used. Compound **3b** was obtained as a white solid. Yield: 47 mg (42%).

#### 3.1.5. General Procedure for the Preparation of Thiosemicarbazones **4a–p**, **5a–c**

To a solution of thiosemicarbazides **3a–c** (200 mg, 1.0 equiv.) in EtOH (5 mL) was added the corresponding aldehyde (1.0 equiv.) and concentrated H_2_SO_4_ (1 drop). The mixture was refluxed for 4 h, giving a precipitate that was filtered and washed with cold EtOH. Title compounds were crystalline solids, stable at rt in solid state and in solution (DMSO). They were soluble in DMSO at a 40 mM concentration.

1-Phenylmethylene-4-(4′-sulfonamidophenyl)-3-thiosemicarbazone (**4a**). Thiosemicarbazide **3a** (200 mg, 0.81 mmol) and benzaldehyde (84 µL, 0.81 mmol, 1.0 equiv.) were used. Compound **4a** was obtained as a white solid. Yield: 192 mg (71%); spectroscopic data are in agreement with those reported [59].

1-(4′-Methoxyphenylmethylene)-4-(4″-sulfonamidophenyl)-3-thiosemicarbazone (**4b**). Thiosemicarbazide **3a** (200 mg, 0.81 mmol) and 4-methoxybenzaldehyde (98 µL, 0,81 mmol, 1.0 equiv.) were used. Compound **4b** was obtained as a white solid. Yield: 138 mg (47%). Mp: 211 °C; 1H-NMR (300 MHz, DMSO-*d*_6_) δ 11.92 (s, 1H, NH), 10.23 (s, 1H, Ar-NH), 8.16 (s, 1H, N=CH) 7.85 (m, 6H, Ar-H), 7.36 (s, 2H, NH_2_), 7.03 (m, 2H, Ar-H), 3.86 (s, 3H, CH_3_) ppm; ^13^C-NMR (125.7 MHz, DMSO-*d*_6_) δ 175.5 (CS), 161.2 (C-4′), 143.7 (C=N), 142.3 (C-4), 140.5 (C-1), 140.3 (C-1) 129.6 (C-2, C-6), 126.5 (C-1′) 125.8 (C-3′, C-5′), 125.3 (C-2′, C-6′) 114.40 (C-3, C-5), 55.5 (CH_3_) ppm. HRESI-MS *m*/*z* calcd. for C_15_H_17_N_4_O_3_S_2_ ([M+H]^+^): 365.0737, found: 365.0731.

1-(4′-Fluorophenylmethylene)-4-(4″-sulfonamidophenyl)-3-thiosemicarbazone (**4c**). Thiosemicarbazide **3a** (200 mg, 0.81 mmol) and 4-fluorobenzaldehyde (87 µL, 0.81 mmol, 1.0 equiv.) were used. Compound **4c** was obtained as a white solid. Yield: 215 mg (76%). Mp: 220–222 °C; ^1^H-NMR (300MHz, DMSO-*d*_6_) δ 11.99 (s, 1H, NH), 10.29 (s, 1H, Ar-NH), 8.18 (s, 1H, N=CH) 7.98 (m, 2H, Ar-H) 7.84 (m, 4H, Ar-H), 7.32 (m, 4H, Ar-H, NH2) ppm; ^13^C-NMR (75.5 MHz, DMSO-*d*_6_) δ 176.0 (C=S), 163.2 (d, ^1^*J*_C,H_ = 247.6 Hz, C-4″), 144.4, 142.1, 140.3 (CH=N, SO_2_NH_2_-Ar-C*p*, NH-Ar-C*ipso*), 130.5 (d, ^4^*J*_C,H_ = 2.9, Hz, C-1″), 130.0 (d, ^3^*J*_C,H_= 8.6 Hz, C-2″/C-6″), 125.6, 125.3 (Ar-C), 115.7 (d, ^2^*J*_C,H_ = 21.9 Hz, C-3″/C-5″) ppm; HRESI-MS *m*/*z* calcd. for C_14_H_13_FN_4_O_3_S_2_ ([M+H]^+^): 353.0537, found: 353.0536.

1-(4′-Chlorophenylmethylene)-4-(4″-sulfonamidophenyl)-3-thiosemicarbazone (**4d**). Thiosemicarbazide **3a** (200 mg, 0.81 mmol) and 4-chlorobenzaldehyde (114 mg, 0,81 mmol, 1.0 equiv.) were used. Compound **4d** was obtained as a white solid. Yield: 223 mg (75%); spectroscopic data are in agreement with those reported [59].

1-(4′-Bromophenylmethylene)-4-(4″-sulfonamidophenyl)-3-thiosemicarbazone (**4e**). Thiosemicarbazide **3a** (200 mg, 0.81 mmol) and 4-bromobenzaldehyde (87 µL, 0.81 mmol, 1.0 equiv.) were used. Compound **4e** was obtained as a white solid. Yield: 236 mg (71%); spectroscopic data are in agreement with those reported [59].

1-(4′-Nitrophenylmethylene)-4-(4″-sulfonamidophenyl)-3-thiosemicarbazone (**4f**). Thiosemicarbazide **3a** (200 mg, 0.81 mmol) and 4-nitrobenzaldehyde (128 mg, 0.81 mmol, 1.0 equiv.) were used. Compound **4f** was obtained as a yellow solid. Yield: 218 mg (72%). Mp: 238 °C; ^1^H-NMR (300 MHz, DMSO-*d*_6_) δ 12.27 (s, 1H, NH), 10.46 (s, 1H, Ar-NH), 8.26 (m, 2H, Ar-H), 8.26 (s, 1H, N=CH), 8.20 (m, 2H, Ar-H), 7.80 (m, 4H, Ar-H), 7.35 (brs, 2H, NH_2_) ppm; ^13^C-NMR (125.7 MHz, DMSO-*d*_6_) δ 176.3 (CS), 147.7 (C-4″), 141.8 (C=N), 140.8, 140.5, 140.2, 128.5, 125.6, 123.7 (Ar-C) ppm; HRESI-MS *m*/*z* calcd. For C_14_H_13_N_5_NaO_4_S_2_ ([M+Na]^+^): 402.0301, found: 402.0296.

1-Phenylmethylene-4-(3″-sulfonamidophenyl)-3-thiosemicarbazone (**4g**). Thiosemicarbazide **3b** (200 mg, 0.81 mmol) and benzaldehyde (84 µL, 0.81 mmol, 1.0 equiv.) were used. Compound **4g** was obtained as a white solid. Yield: 197 mg (68%); spectroscopic data are in agreement with those reported [59].

1-(4′-Methoxyphenylmethylene)-4-(3″-sulfonamidophenyl)-3-thiosemicarbazone (**4h**). Thiosemicarbazide **3b** (200 mg, 0.81 mmol) and 4-methoxybenzaldehyde (98 µL, 0,81 mmol, 1.0 equiv.) were used. Compound **4h** was obtained as a white solid. Yield: 140 mg (48%). Mp: 184 °C; ^1^H-NMR (300 MHz, DMSO-*d*_6_) δ 11.84 (s, 1H, NH), 10.2 (s, 1H, Ar-NH), 8.12 (s, 1H, N=CH), 8.05 (brt, 1H, *J*_H,H_ = 1.9 Hz, H-2″), 7.86 (m, 2H, H-2′, H-6′), 7.84 (m, 1H, Ar-H), 7.64 (dt, 1H, *J*_H,H_ = 1.4 Hz, *J*_H,H_ = 8.4 Hz, Ar-H), 7.54 (t, 1H, *J*_H,H_ = 7.9 Hz, Ar-H), 7.40 (s, 2H, NH_2_), 6.99 (m, 2H, H-3′, H-5′), 3.81 (s, 1H, OMe) ppm; ^13^C-NMR (75.5 MHz, DMSO-d6) δ 176.1 (CS), 161.1 (C-4′), 144.4 (C=N), 143.8 (C-3″), 140.0 (C-1″), 129.8 (C-2′/C-6′), 129.6, 128.9, 126.9, 123.2, 122.6 (Ar-C), 114.64 (C-3′/C-5′), 55.8 (OMe) ppm; HRESI-MS *m*/*z* calcd. for C_15_H_16_N_4_NaO_3_S_2_ ([M+Na]+): 387.0556, found: 387.0547.

1-(4′-Fluorophenylmethylene)-4-(3″-sulfonamidophenyl)-3-thiosemicarbazone (**4i**). Thiosemicarbazide **3b** (200 mg, 0.81 mmol) and 4-fluorobenzaldehyde (87 µL, 0,81 mmol, 1.0 eq.) were used. Compound **4i** was obtained as a white solid. Yield: 198 mg (70%); spectroscopic data are in agreement with those reported [59].

1-(4′-Chlorophenylmethylene)-4-(3″-sulfonamidophenyl)-3-thiosemicarbazone (**4j**). Thiosemicarbazide **3b** (200 mg, 0.81 mmol) and 4-chlorobenzaldehyde (114 mg, 0,81 mmol, 1.0 equiv.) were used. Compound **4j** was obtained as a white solid. Yield: 225 mg (76%). Mp: 229 °C; ^1^H-NMR (300 MHz, DMSO-d_6_) δ 11.99 (s, 1H, NH), 10.35 (s, 1H, Ar-NH), 8.15 (s, 1H, N=CH), 8.04 (brt, 1H, J_H,H_ = 1.8 Hz, H-2″), 7.97 (m, 2H, H-2′, H-6′), 7.81 (brd, 1H, J_H,H_ = 8.0 Hz, Ar-H), 7.65 (brd, 1H, J_H,H_ = 7.9 Hz, Ar-H), 7.60–7.49 (m, 3H, Ar-H), 7.38 (brs, 2H, NH_2_) ppm; ^13^C-NMR (75.5 MHz, DMSO-d_6_) δ 176.2 (CS), 144.0, 142.0, 139.5 (CH=N, C-1″, C-3″), 134.6 (C-1′), 132.9, 129.3, 128.7, 128.5, 122.9, 122.4 (Ar-C) ppm; HRESI-MS calcd. for C_14_H_13_^35^ClN_4_NaO_2_S_2_ ([M+Na]^+^): 391.0061, found: 391.0057.

1-(4′-Bromophenylmethylene)-4-(3″-sulfonamidophenyl)-3-thiosemicarbazone (**4k**). Thiosemicarbazide **3b** (200 mg, 0.81 mmol) and 4-bromobenzaldehyde (87 µL, 0,81 mmol, 1.0 equiv.) were used. Compound **4k** was obtained as a white solid. Yield: 250 mg (76%); spectroscopic data are in agreement with those reported [59].

1-Phenylmethylene-4-[2′-(4″-sulfonamido)phenyl]ethyl-3-thiosemicarbazone (**4l**). Thiosemicarbazide **3c** (200 mg, 0.73 mmol) and benzaldehyde (74 µL, 0.73 mmol, 1.0 equiv.) were used. Compound **4l** was obtained as a white solid. Yield: 133 mg (50%). Mp: 228 °C; ^1^H-NMR (300 MHz, DMSO-d_6_) δ 11.56 (s, 1H, NH), 8.59 (t, 1H, J_H,H_ = 6.3 Hz, NH-CH_2_), 8.10 (s, 1H, N=CH), 7.80 (m, 4H, Ar-H), 7.45 (m, 5H, Ar-H), 7.28 (s, 2H, NH_2_), 3.81 (brq, 2H, J_H,H_ = 6.5 Hz, NH-CH_2_), 3.02 (t, 2H, J_H,H_ = 6.5 Hz, CH_2_-Ph) ppm; ^13^C-NMR (125.7 MHz, DMSO-d_6_) δ 177.1 (CS), 143.5, 142.2, 142.1, (CH=N, C-4″, C-1″), 134.2 (C-1, Ph), 129.9, 129.1, 128.7, 127.2, 125,9 (Ar-C), 44.6 (CH2-NH), 34.6 (CH_2_-Ar) ppm; HRESI-MS *m*/*z* calcd. for C_16_H_18_N_4_NaO_2_S_2_ ([M+Na]^+^): 385.0763, found: 385.0761.

1-(4′-Methoxyphenylmethylene)-4-[2″-(4‴-sulfonamido)phenyl]ethyl-3-thiosemicarbazone (**4m**). Thiosemicarbazide **3c** (200 mg, 0.73 mmol) and 4-methoxybenzaldehyde (89 µL, 0.73 mmol, 1.0 equiv.) were used. Compound **4m** was obtained as a white solid. Yield: 186 mg (65%). Mp: 174 °C; ^1^H-NMR (300 MHz, DMSO-d_6_) δ 11.38 (s, 1H, NH), 8.47 (t, 1H, J_H,H_ = 6.0 Hz, NH-CH_2_), 8.00 (s, 1H, N=CH), 7.77 (m, 2H, Ar-H), 7.70 (m, 2H, Ar-H), 7.45 (m, 2H, Ar-H), 7.27 (s, NH_2_), 6.98 (m, 2H, Ar-H), 3.80 (s, 3H, OMe), 3.80 (m, 2H, CH_2_-NH), 3.00 (t, 2H, J_H,H_ = 8.0 Hz, CH_2_-Ar) ppm; ^13^C-NMR (75.5 MHz, DMSO-d_6_) δ 176.8 (CS), 160.7 (C-4′), 143.5, 142.1, 142.0 (N=CH, C-4‴, C-1‴), 129.1, 128.8, 126.7, 125.8, 114.2 (Ar-C), 55.3 (OMe), 44.4 (CH_2_-NH), 34.8 (Ar-CH_2_) ppm; HRESI-MS *m*/*z* calcd. for C_17_H_20_N_4_NaO_3_S_2_ ([M+Na]^+^): 415.0869, found: 415.0862.

1-(4′-Fluorophenylmethylene)-4-[2″-(4‴-sulfonamido)phenyl]ethyl-3-thiosemicarbazone (**4n**). Thiosemicarbazide **3c** (200 mg, 0.73 mmol) and 4-fluorobenzaldehyde (78 µL, 0.73 mmol, 1.0 equiv.) were used. Compound **4n** was obtained as a white solid. Yield: 205 mg (74%). Mp: 242 °C; ^1^H-NMR (300 MHz, DMSO-d_6_) δ 11.52 (s, 1H, NH), 8.61 (t, 1H, J_H,H_ = 5.6 Hz, NH-CH_2_), 8.05 (s, 1H, N=CH), 7.84 (m, 2H, Ar-H), 7.78 (m, 2H, Ar-H), 7.46 (m, 2H, Ar-H), 7.28 (m, 4H, Ar-H, NH_2_), 3.80 (q, 2H, J_H,H_ = 7.0 Hz, CH_2_-NH), 3.01 (t, 1H, J_H,H_ = 7.0 Hz, CH_2_-Ar) ppm; ^13^C-NMR (125.7 MHz, DMSO-d_6_) δ 177.1 (C=S), 163.0 (d, ^1^J_H,H_ = 247.1 Hz, C-4′), 143.5, 142.2, 140.9, (CH=N, C-4‴, C-1‴), 130.8 (d, ^4^J_H,H_ = 3.7 Hz, C-1′), 129.4 (d, ^3^J_H,H_= 7.6 Hz, C-2′/C-6′), 129.1, 125.9 (Ar-C), 115.8 (d, ^2^J_H,H_ = 21.7 Hz, C-3′/C-5′), 44.6 (CH_2_-NH), 34.6 (CH_2_-Ar), ppm; HRESI-MS *m*/*z* calcd. for C_16_H_17_FN_4_NaO_2_S_2_ ([M+Na]^+^): 403.0669, found: 403.0670.

1-(4′-Chlorophenylmethylene)-4-[2″-(4‴-sulfonamido)pheny]ethyl-3-thiosemicarbazone (**4o**). Thiosemicarbazide **3c** (200 mg, 0.73 mmol) and 4-chlorobenzaldehyde (103 mg, 0.73 mmol, 1.0 equiv.) were used. Compound **4o** was obtained as a white solid. Yield: 150 mg (52%). Mp: 235 °C; ^1^H-NMR (300 MHz, DMSO-d_6_) δ 11.57 (s, 1H, NH), 8.65 (t, 1H, J_H,H_ = 5.9 Hz, NH-CH_2_), 8.05 (s, 1H, N=CH) 7.80 (m, 4H, Ar-H), 7.48 (m, 4H, Ar-H), 7.29 (s, 2H, NH_2_), 3.81 (brq, 2H, J_H,H_ = 6.6 Hz, CH_2_-NH), 3.02 (t, 2H, J_H,H_ = 7.1 Hz, CH_2_-Ar) ppm; ^13^C-NMR (125.7 MHz, DMSO-d_6_) δ 177.1 (CS), 143.5, 142.2, 140.7, (CH=N, C-4‴, C-1‴), 134.3, 133.2, 129.1, 128.9, 128.8, 125.9 (Ar-C), 44.6 (CH_2_-NH), 34.6 (CH_2_-Ar) ppm; HRESI-MS *m*/*z* calcd. for C_16_H_17_^35^ClN_4_NaO_2_S_2_ ([M+Na]^+^): 419.0374, found: 419.0367.

1-(4′-Bromophenylmethylene)-4-[2″-(4‴-sulfonamido)phenyl]ethyl-3-thiosemicarbazone (**4p**). Thiosemicarbazide **3c** (200 mg, 0.73 mmol) and 4-bromobenzaldehyde (78 µL, 0.82 mmol, 1.0 equiv.) were used. Compound **4p** was obtained as a white solid. Yield: 187 mg (58%). Mp: 244 °C; ^1^H-NMR (300 MHz, DMSO-d_6_) δ 11.57 (s, 1H, NH), 8.64 (t, 1H, J_H,H_ = 5.8 Hz, NH-CH_2_), 8.03 (s, 1H, N=CH), 7.76 (m, 4H, Ar-H), 7.63 (m, 2H, Ar-H’), 7.46 (m, 2H, Ar-H), 7.28 (s, 2H, NH_2_), 3.80 (q, 2H, J_H,H_ = 6.5 Hz, CH_2_-NH), 3.02 (t, 2H, J_H,H_ = 7.0 Hz, CH_2_-Ar) ppm; ^13^C-NMR (75.5 MHz, DMSO-d_6_) δ 177.6 (CS), 143.5, 142.2, 140.8 (N=CH, C-4‴, C-1‴), 133.9, 132.1, 129.5, 126.3, 123.5 (Ar-C), 44.6 (CH_2_-NH), 34.6 (CH_2_-Ar); HRESI-MS *m*/*z* calcd. for C_16_H_18_^79^BrN_4_O_2_S_2_ ([M+H]^+^): 441.0049, found: 441.0049; *m*/*z* calcd. for C_16_H_18_^81^BrN_4_O_2_S_2_ ([M+H]^+^): 443.0029, found: 443.0026.

1-(Pyridin-2′-ylmethylene)-4-(4″-sulfonamidophenyl)-3-thiosemicarbazone (**5a**). Thiosemicarbazide **3a** (200 mg, 0.81 mmol) and pyridine-2-carbaldehyde (74 µL, 0.81mmol, 1.0 equiv.) were used. Compound **5a** was obtained as a yellow solid. Yield: 84 mg (31%). Mp: 178 °C; ^1^H-NMR (300 MHz, DMSO-d_6_) δ 12.48 (s, 1H, NH), 10.52 (s, 1H, Ar-NH), 8.76 (brd, 1H, J_5′,6′_ = 5.1 Hz, H-6′), 8.47 (d, 1H, J_3′,4′_ = 8.4 Hz, H-3′), 8.23 (s, 1H, N=CH), 8.18 (m, 1H, H-4′), 7.82 (s, 4H, Ar-Ho, Ar-Hm), 7.67 (m, 1H, H-5′), 7.36 (s, 2H, NH_2_) ppm; ^13^C-NMR (125.7 MHz, DMSO-d_6_) δ 176.7 (CS), 150.2 (C-2′), 146.7 (C-6′), 141.9 (C=N), 140.9, 139.2, 126.0, 125.8, 125.6, 123.3, 123.3 (Ar-C) ppm; HRESI-MS *m*/*z* calcd. for C_13_H_13_N_5_NaO_2_S_2_ ([M+Na]^+^): 358.0403, found: 358.0402.

1-(Pyridin-2′-ylmethylene)-4-(3″-sulfonamidophenyl)-3-thiosemicarbazone (**5b**). Thiosemicarbazide **3b** (200 mg, 0.73 mmol) and pyridine-2-carbaldehyde (74 µL, 0.81mmol, 1.0 equiv.) were used. Compound **5b** was obtained as a yellow solid. Yield: 111 mg (41%). Mp: 186 °C (dec.); ^1^H-NMR (300 MHz, DMSO-d_6_) δ 12.34 (s, 1H, NH), 10.51 (s, 1H, Ar-NH), 8.68 (brd, 1H, J_5′,6′_ = 5.1 Hz, H-6″), 8.47 (d, 1H, J_3′,4′_ = 8.3 Hz, H-3′), 8.21 (s, 1H, N=CH), 8.07 (t, 1H, J_H,H_ = 7.7 Hz, Ar-H), 8.04 (brt, 1H, J_2″,4″_= J_2″,6″_ = 1.8 Hz, H-2″), 7.82 (brd, 1H, J_H,H_ = 7.8 Hz, Ar-H), 7.68 (dt, 1H, J_H,H_ = 1.3 Hz, J_H,H_ = 7.8 Hz, Ar-H), 7.58 (m, 2H, Ar-H), 7.41 (s, 2H, NH_2_) ppm; ^13^C-NMR (75.5 MHz, DMSO-d_6_) δ 176.7 (CS), 150.9 (C-2′), 147.2 (C-3″), 144.2 (C=N), 140.1, 139.6, 139.2, 129.4, 128.7, 125.1, 122.9, 122.7, 122.3 (Ar-C) ppm; HRESI-MS *m*/*z* calcd. For C_13_H_13_N_5_NaO_2_S_2_ ([M+Na]^+^): 358.0403, found: 358.0399.

1-(Pyridin-2′-ylmethylene)-4-[2″-(4‴-sulfonamidophenyl)]ethyl-3-thiosemicarbazone (**5c**). Thiosemicarbazide **3c** (200 mg, 0.73 mmol, 1.0 equiv.) and pyridine-2-carbaldehyde (67 µL, 0.73 mmol) were used. Compound **5b** was obtained as a yellow solid. Yield: 111 mg (42%). Mp: 225 °C; ^1^H-NMR (300 MHz, DMSO-d_6_) δ 12.11 (s, 1H, NH), 8.99 (t, 1H, J_H,H_ = 6.0 Hz, NH-CH_2_), 8.77 (brd, 1H, J_5′,6′_ = 5.1 Hz, H-6′), 8.35 (brd, 1H, J_3′,4′_ = 8.4 Hz, H-3′), 8.26 (brtd, 1H, J_4′,6′_ = 1.4 Hz, J_4′,5′_= 7.8 Hz, H-4′), 8.13 (s, 1H, N=CH), 7.77 (m, 2H, Ar-H), 7.72 (m, 1H, H-5′), 7.46 (m, 2H, Ar-H), 7.28 (brs, 2H, NH_2_), 3.84 (q, 2H, J_H,H_ = 6.5 Hz, NH-CH_2_), 3.03 (t, 1H, J_H,H_ = 7.1 Hz, CH_2_-Ar) ppm; ^13^C-NMR (75.5 MHz, DMSO-d_6_) δ 178.0 (CS), 155.1 (C-2′), 148.3 (C-6′), 144.4 (C=N), 143.7, 143.3, 143.3, 142.2, 134.4, 129.2, 125.8, 124.3 (Ar-C), 44.8 (CH_2_-NH), 34.3 (CH_2_-Ar) ppm; HRESI-MS *m*/*z* calcd. for C_15_H_17_N_5_NaO_2_S_2_ ([M+Na]^+^): 386.0716, found: 386.0720.

### 3.2. CA Inhibition Assays

An applied photophysics stopped-flow instrument was used for assaying the CA-catalyzed CO_2_ hydration activity [18]. Phenol red (at 0.2 mM) was used as an indicator, working at the absorbance maximum of 557 nm, with 20 mM Hepes (pH 7.4) and 20 mM Na_2_SO_4_ (for maintaining constant ionic strength), following the initial rates of the CA-catalyzed CO_2_ hydration reaction for 10–100 s. The CO_2_ concentrations ranged from 1.7 to 17 mM for the determination of the kinetic parameters and inhibition constants. For each inhibitor, at least six traces of the initial 5–10% of the reaction were used for determining the initial rate. The uncatalyzed rates were determined in the same manner and subtracted from the total observed rates. Stock solutions of inhibitor (10 mM) were prepared in distilled–deionized water, and appropriate dilutions were done thereafter with distilled–deionized water. AAZ was used as a positive control. Inhibitor and enzyme solutions were preincubated together for 15 min at room temperature before assay in order to allow for the formation of the E–I complex. The inhibition constants were obtained by nonlinear least-squares methods using PRISM 3 and the Cheng–Prusoff equation and represent the mean from at least three different determinations. All CA isoforms were recombinant ones (5–12 nM), obtained in-house.

### 3.3. Docking Simulations

The crystallographic structure of CAIX and CAXII with 1.82 Å resolution (PDB ID: 5FL4) and 1.38 Å resolution (PDB ID: 4HT2), respectively, were obtained from the Protein Data Bank [60]. Docking simulations were performed in MOE Software v2019.01 (Chemical Computing Group, Montreal, QC, Canada). Protein optimization was performed following the QuickPrep protocol. Briefly, crystallographic artifacts, non-bonded ligands, and excess copies of the protein are removed. Water molecules were removed except for the ones at a maximum distance of 4.5 Å from the active site. Ligands were built, hydrogens added, and geometry optimized through energy minimization. During docking simulations, ligands were placed in the grid of the co-crystallized ligand. In the placement stage, energy binding calculations used the Triangle Matcher algorithm with the London dG scoring scheme. In the refinement stage, the receptor was kept rigid, and the GBVI/WSA dg scoring scheme was used.

### 3.4. Metal Complexation Assays

The complexation of **5b** with different metal chlorides (NaCl, KCl, FeCl_2_, FeCl_3_, ZnCl_2_, and CuCl_2_) was investigated using UV–Vis spectroscopy (Jasco V-360). The stock solutions of the salts (10^−2^ M) and the ligand (2.5 mM) were prepared in pure DMSO and sonicated when necessary. To 20 µL of a 2.5 mM solution of **5b** were added different aliquots of the metal chlorides solutions up to a total volume of 2 mL in quartz cuvettes. The following ligand–metal ratios were used: 1:0.25, 1:0.5, 1:1, 1:2, 1:5, and 1:10. The absorbance was monitored at 25 °C in the range 280–440 nm, either with or without incubation. A blank solution without **5b** was also prepared for each concentration of the salt, and its spectrum was subtracted from the ligand–salt spectrum.

### 3.5. Antiproliferative Assays

#### 3.5.1. Cell Lines and Culture

The human cancer cell lines A549, HBL-100, and T-47D, as well as HeLa, were provided by Dr. Raimundo Freire (Hospital Universitario de Canarias, Tenerife, Canary Islands). The lung cancer cell lines SW1573 and WiDr were provided by Prof. G. J. Peters (VU University Medical Center, Amsterdam, The Netherlands). Cells were grown in RPMI-1640 medium containing 5% fetal bovine serum (FBS), 2 mM l-glutamine, 100 U/mL of penicillin G, and 0.1 mg/mL of streptomycin at 37 °C in a 95% humidified atmosphere of 5% CO_2_. Cells were maintained in culture in 60 mm cell culture dishes in growth medium (10 mL) and passaged twice weekly.

#### 3.5.2. Antiproliferative Tests

The antiproliferative activity of compounds was tested using our implementation of the protocol of the National Cancer Institute (NCI) of the USA. The following seeding densities (cells per well) were used: 2500 (A549, HBL-100, HeLa, and SW1573) and 5000 (T-47D and WiDr). Stock solutions of inhibitors (40 mM) were prepared in pure DMSO (400 times the maximum test concentration). For each test compound, the cells were exposed for a period of 48 h to serial decimal dilutions in cell culture medium of the test compounds (0.001–100 μM). For each product, GI_50_ values were calculated according to the NCI formulas (n = 3; data are expressed as mean ± SD).

#### 3.5.3. Cell Morphology

The CX-A imaging platform microscope (Nanolive SA, Lausanne, Switzerland) was used to measure refractive indices, creating a holotomographic 3D image of the cells. SW1573 cells were seeded onto 35 mm cell culture imaging dishes (IBIDI GmbH, Gräfelfing, Germany) at a density of 50.000 cells/well. On the next day, treated cells were exposed to the test compounds right before the acquisition of the images. Image data were transferred to FIJI software v2.9.0 (NIH, USA) for image analysis. EVE software v2.2.1.2162 (Nanolive S.A., Tolochenaz, Switzerland)) was used for the analysis of the refractive indices and calculation of the phenotypic parameters.

## 4. Conclusions

In conclusion, we have successfully designed multifunctional antiproliferative agents by combining aryl sulfonamides, which act as pharmacophores for CA inhibition, with a thiosemicarbazone moiety that acts as a metal chelator. Pyridine-2-carbaldehyde thiosemicarbazone **5b**, with the sulfonamido motif at the *meta* position, can be considered as the lead compound. Although the presence of the sulfonamido moiety was found to be deleterious to the antiproliferative activity compared with other α-*N*-heterocyclic thiosemicarbazones reported in the literature, **5b** still preserved good activity (GI_50_ values in the low micromolar range), potent inhibition of CA isoforms associated with tumor progression (IX and XII, *K*_i_ values in the low nanomolar range), and effective chelation of divalent cations Fe^2+^ and Cu^2+^, ensuring a multifactorial mechanism of action. The absence of a direct correlation between CA inhibition and antiproliferative effects suggests that additional biological targets may also be involved. The mode of action of **5b** was further explored using 3D holotomographic microscopy, which revealed delayed tumor cell division and features indicative of apoptosis.

## Figures and Tables

**Table 1:** Inhibition data (*K*_i_, nM) of compounds **4** and **5** against human CAs I, II, IX, and XII ^[a]^ using AAZ as a standard drug.

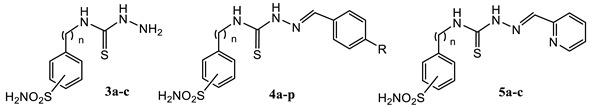						
Compound	CA I	CA II	CA IX	CA XII	Selectivity Ratio I/IX//II/IX	Selectivity Ratio I/XII//II/XII
**3a** (n = 0, *p*-)	327	7600	133	68	2.5//57.1	4.8//111.8
**3b** (n = 0, *m-*)	1457	439	185	116	7.9//2.4	12.6//3.8
**3c** (n = 2, *p*-)	79.0	8.51	13.0	5.02	6.1//0.65	15.7//1.7
**4a** (n = 0, *p*-, R = H)	504	23.7	16.1	8.38	31.3//1.5	60.1//2.8
**4b** (n = 0, *p*-, R = OMe)	215	78	91	49	2.4//0.86	4.4//1.6
**4c** (n = 0, *p*-, R = F)	196	44.6	147	51.8	1.3//0.30	3.8//0.86
**4d** (n = 0, *p*-, R = Cl)	485	48.8	27.4	12.1	17.7//1.8	40.1//4.0
**4e** (n = 0, *p*-, R = Br)	574	46.8	23.3	13.6	24.6//2.0	42.2//3.4
**4f** (n = 0, *p*-, R = NO_2_)	845	820	477	288	1.8//1.7	2.9//2.8
**4g** (n = 0, *m*-, R = H)	876	60.5	74.3	50.9	11.8//0.81	17.2//1.2
**4h** (n = 0, *m*-, R = OMe)	748	39.4	387	2.3	1.9//0.10	325//17.1
**4i** (n = 0, *m*-, R = F)	496	97.1	89.3	49.3	5.6//1.1	10.1//2.0
**4j** (n = 0, *m*-, R = Cl)	5186	312	217	85.4	23.9//1.4	60.7//3.7
**4k** (n = 0, *m*-, R = Br)	5337	531	206	91.3	25.9//2.6	58.5//5.8
**4l** (n = 2, *p*-, R = H)	568	70.2	30.8	9.12	18.4//2.3	62.3//7.7
**4m** (n = 2, *p*-, R = OMe)	3479	321	164	35.0	21.2//2.0	99.4//9.2
**4n** (n = 2, *p*-, R = F)	2485	590	300	48.5	8.3//2.0	51.2//12.2
**4o** (n = 2, *p*-, R = Cl)	6217	773	408	143	15.2//1.9	43.4//2.9
**4p** (n = 2, *p*-, R = Br)	6092	813	499	123	12.2//1.6	49.5//6.6
**5a** (n = 0, *p*-)	207	156.6	351.9	4.9	0.59//0.45	42.2//31.9
**5b** (n = 0, *m*-)	538	2.5	4.9	5.6	109.8//0.49	96.1//0.43
**5c** (n = 2, *p*-)	270	83.9	28.5	9.3	9.5//2.9	29.0//9.0
**AAZ**	250.0	12.0	25.0	5.7	10.0//0.48	43.9//2.1

^[a]^ Mean from three different assays by a stopped flow technique (errors were in the range of ±5–10% of the reported values).

**Table 2:** Binding energies estimated for compounds **4a** and **5b** in their interaction with CA IX.

Compound	Binding Energy (kcal/mol)
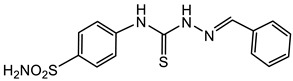 **4a**	−7.93
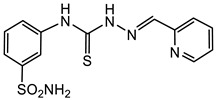 **5b**	−7.45

**Table 3:** Binding energies resulting from the docking simulations of the selected molecules against CA XII. Re-docking score of 6R6Y co-crystallized ligand is also included.

Compound	Binding Energy (kcal/mol)
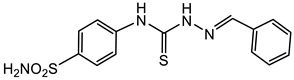 **4a**	−8.20
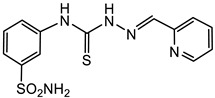 **5b**	−7.68

**Table 4:** GI_50_ values (µM) for the antiproliferative activity of derivatives **4** and **5**.

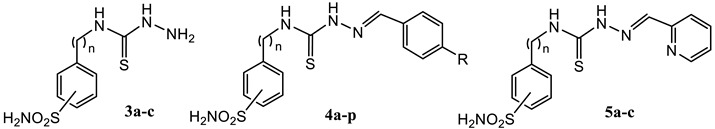						
Compound	A549 (Lung)	HBL-100 (Breast)	HeLa (Cervix)	SW1573 (Lung)	T-47D (Breast)	WiDr (Colon)
**3a** (n = 0, *p*-)	>100					
**3b** (n = 0, *m-*)						
**3c** (n = 2, *p*-)						
**4a** (n = 0, *p*-, R = H)	5.1 ± 3.7	3.0 ± 1.6	2.3 ± 0.2	2.9 ± 1.4	2.6 ± 0.1	4.5 ± 1.4
**4b** (n = 0, *p*-, R = OMe)	>100		64 ± 23	>100		
**4c** (n = 0, *p*-, R = F)	25 ± 5	28 ± 1	7.9 ± 1.1	6.0 ± 0.4	17 ± 1	24 ± 4
**4d** (n = 0, *p*-, R = Cl)	>100					
**4e** (n = 0, *p*-, R = Br)	23 ± 6	24 ± 2	18 ± 1	22 ± 1	36 ± 1	62 ± 12
**4f** (n = 0, *p*-, R = NO_2_)	>100					
**4g** (n = 0, *m*-, R = H)	51 ± 10	>100	61 ± 6	70 ± 1	83 ± 10	62 ± 10
**4h** (n = 0, *m*-, R = OMe)	>100					
**4i** (n = 0, *m*-, R = F)	83 ± 16	>100				79 ± 29
**4j** (n = 0, *m*-, R = Cl)	22 ± 4	42 ± 15	38 ± 3	36 ± 4	59 ± 16	24 ± 8
**4k** (n = 0, *m*-, R = Br)	19 ± 6	40 ± 1	45 ± 5	46 ± 1	>100	
**4l** (n = 2, *p*-, R = H)	90 ± 14	>100		99 ± 1	>100	90 ±14
**4m** (n = 2, *p*-, R = OMe)	97 ± 4	>100				
**4n** (n = 2, *p*-, R = F)	>100					
**4o** (n = 2, *p*-, R = Cl)	62 ± 6	76 ± 32	45 ±16	73 ± 38	>100	>100
**4p** (n = 2, *p*-, R = Br)	21 ± 4	42 ± 8	31 ± 9	53 ± 17	37 ± 8	58 ± 9
**5a** (n = 0, *p*-)	18 ± 2	5.2 ± 2.2	5.2 ± 0.3	4.2 ± 0.1	5.7 ± 2.1	3.8 ± 0.3
**5b** (n = 0, *m*-)	10 ± 2	7.8 ± 2	5.8 ± 0.4	7.1 ± 0.4	5.3 ± 0.7	4.5 ± 0.6
**5c** (n = 2, *p*-)	37 ± 11	>100				
**5-Fluorouracil**	2.2 ± 0.3	4.4 ± 0.7	16 ± 5	3.3 ± 1.2	43 ± 16	49 ± 7
**CDDP**	4.9 ± 0.2	1.9 ± 0.2	1.8 ± 0.5	2.7 ± 0.4	17 ± 3	26 ± 4

## Data Availability

Dataset available on request from the authors.

## Supplementary Materials

The following supporting information can be downloaded at https://www.mdpi.com/article/10.3390/ijms26031225/s1.
