# Supplementary material for: Multifaceted Sulfonamide-Derived Thiosemicarbazones: Combining Metal Chelation and Carbonic Anhydrases Inhibition in Anticancer Therapy
Source: Int J Mol Sci. 2025 Jan 30;26(3):1225. doi: 10.3390/ijms26031225 (PMC11818225; doi:10.3390/ijms26031225)
Supplement: Supplementary file 1 [file ijms-26-01225-s001.zip › Supporting information-Sulfonamides-derived thiosemicarbazones.pdf]

Multifaceted Sulfonamide-Derived Thiosemicarbazones: Combining Metal Chelation and Carbonic Anhydrases Inhibition in Anticancer Therapy

Mónica Martínez-Montiel,[1,2,#] Giulia Arrighi[1,3,#], Paloma Begines,[1,3] Aday González-Bakker ,[4]Adrián Puerta,[4] Miguel X. Fernandes,[4] Penélope Merino-Montiel[2] Sara Montiel-Smith,[2] Alessio Nocentini,[3] Claudiu T. Supuran[3], José M. Padrón[4],José G. Fernández-Bolaños[1], and Óscar López[1,*]

^{1}H and ^{13}C-NMR spectra…………………………………………………………………..................……………………………………………………………….S1–S26

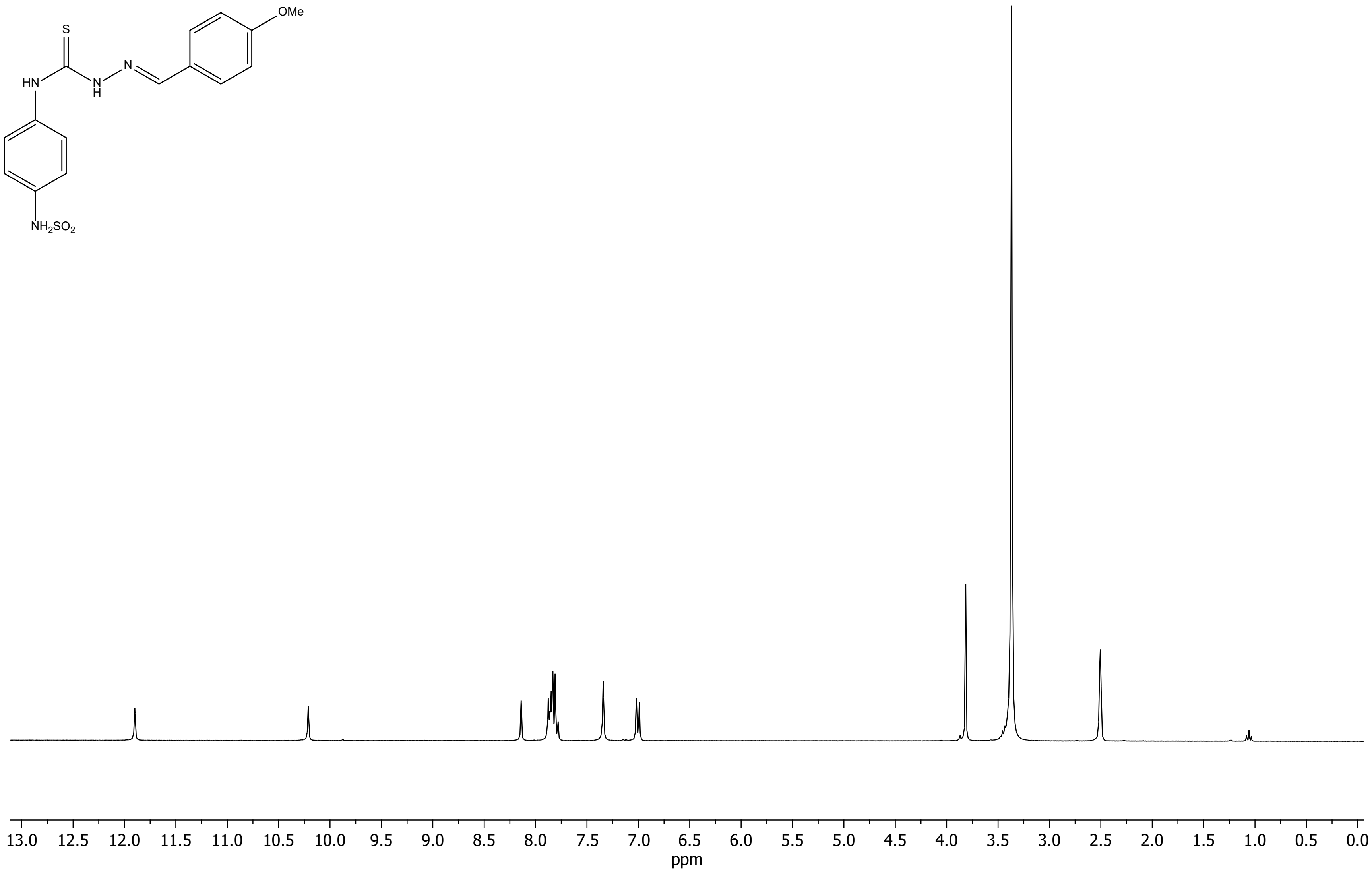

Figure S1. ^{1}H-NMR spectrum of **4b** (300 MHz, DMSO-d_6)

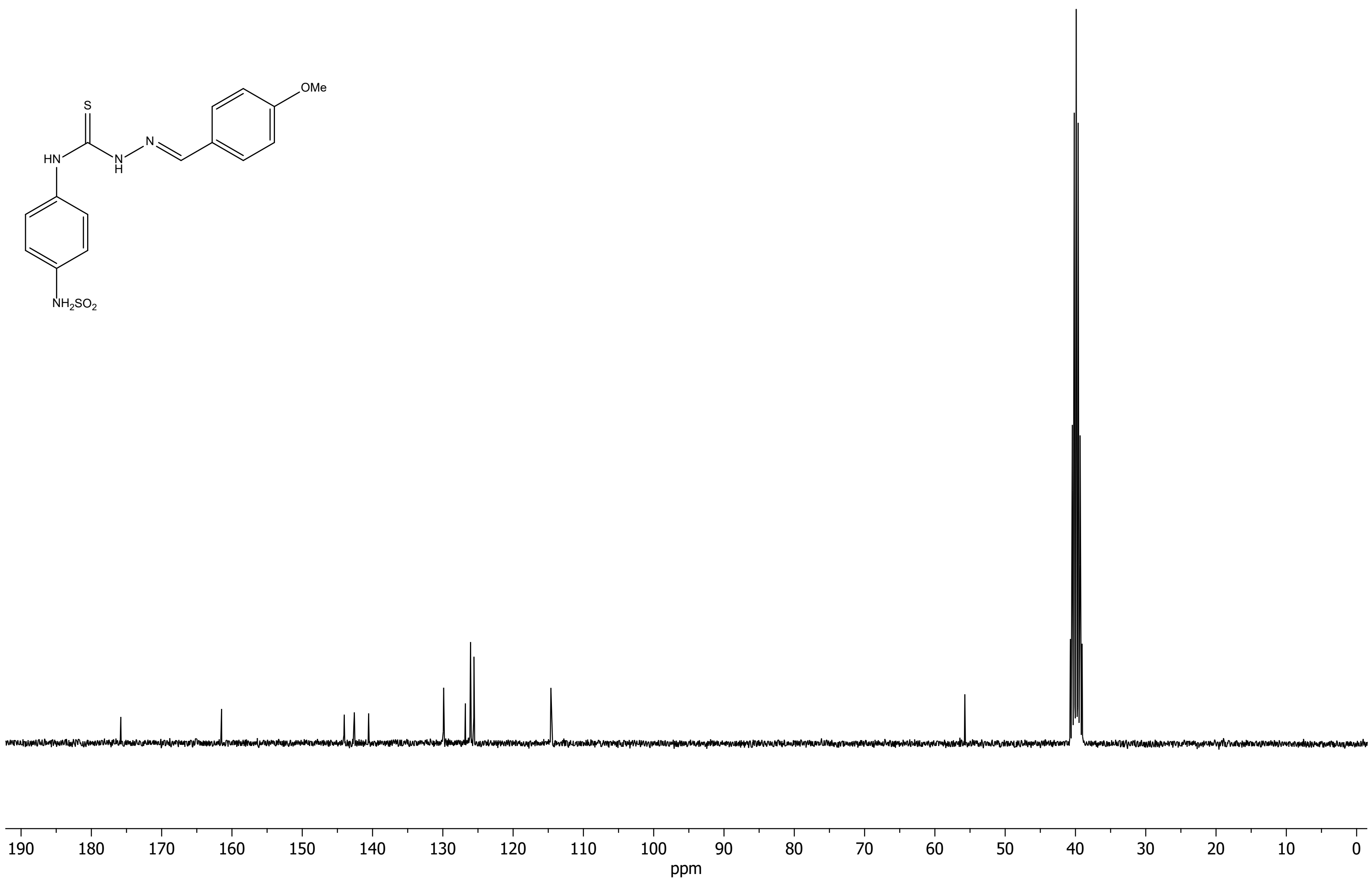

Figure S2. ^{13}C-NMR spectrum of **4b** (125.7 MHz, DMSO-d_6)

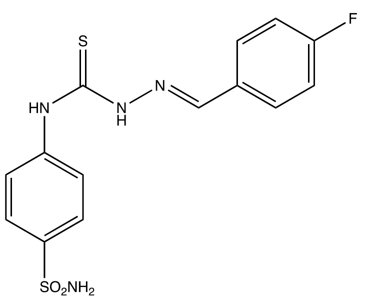

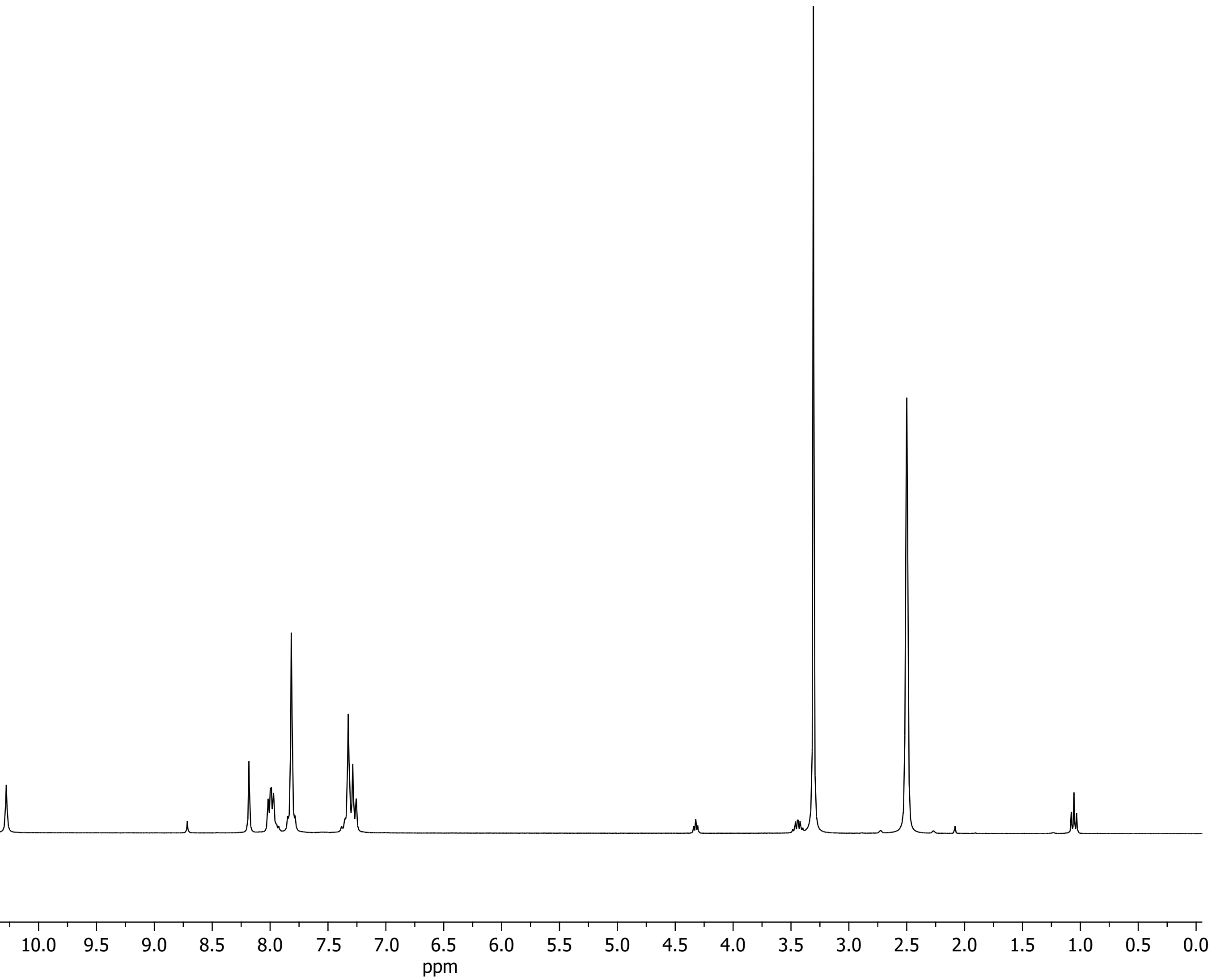

Figure S3. ^{1}H-NMR spectrum of **4c** (300 MHz, DMSO-d_6)

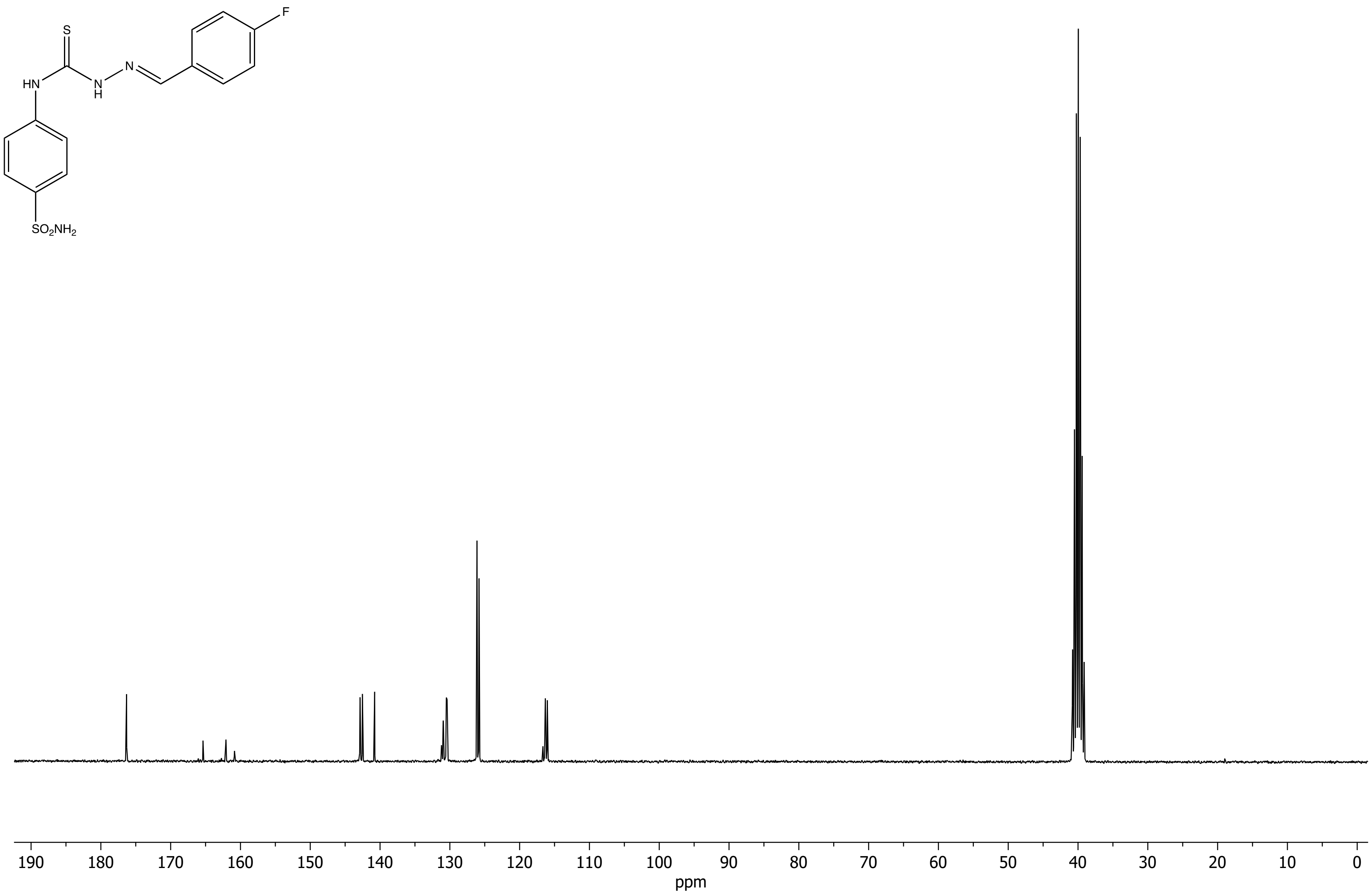

Figure S4. ^{13}C-NMR spectrum of **4c** (75.5 MHz, DMSO-d_6)

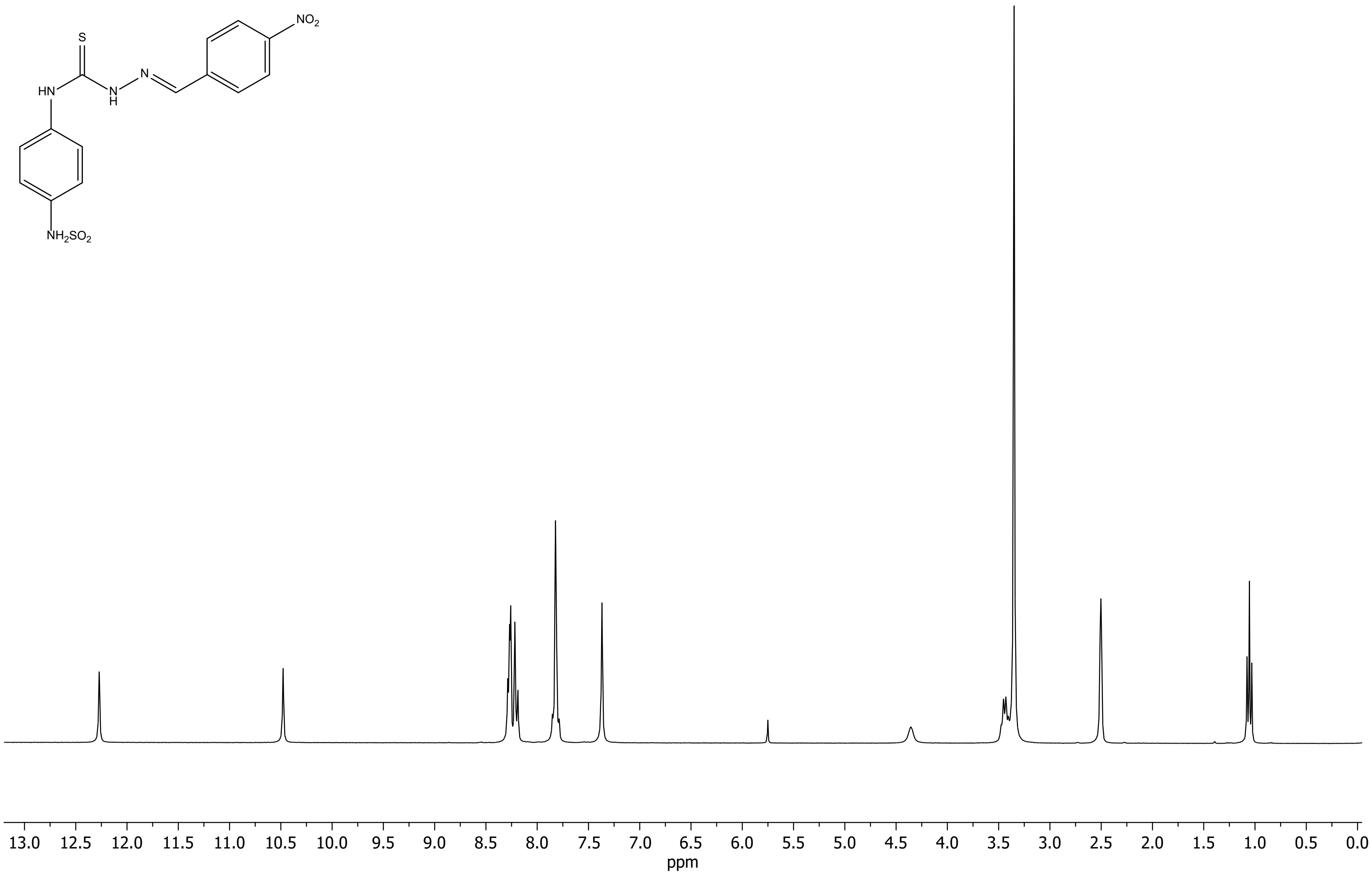

Figure S5. ^{1}H-NMR spectrum of **4f** (300 MHz, DMSO-d_6)

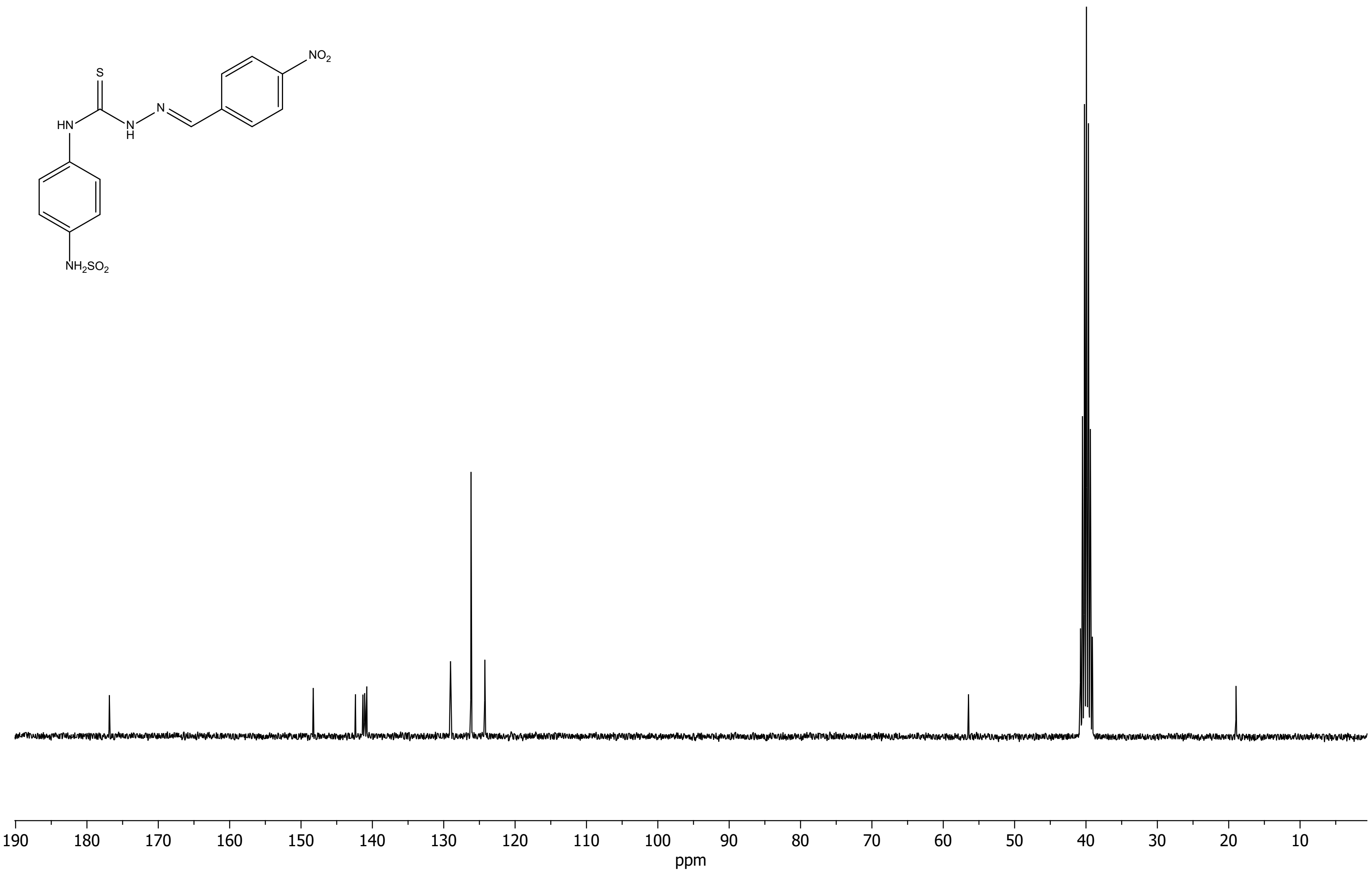

Figure S6. ^{13}C-NMR spectrum of **4f** (125.7 MHz, DMSO-d_6)

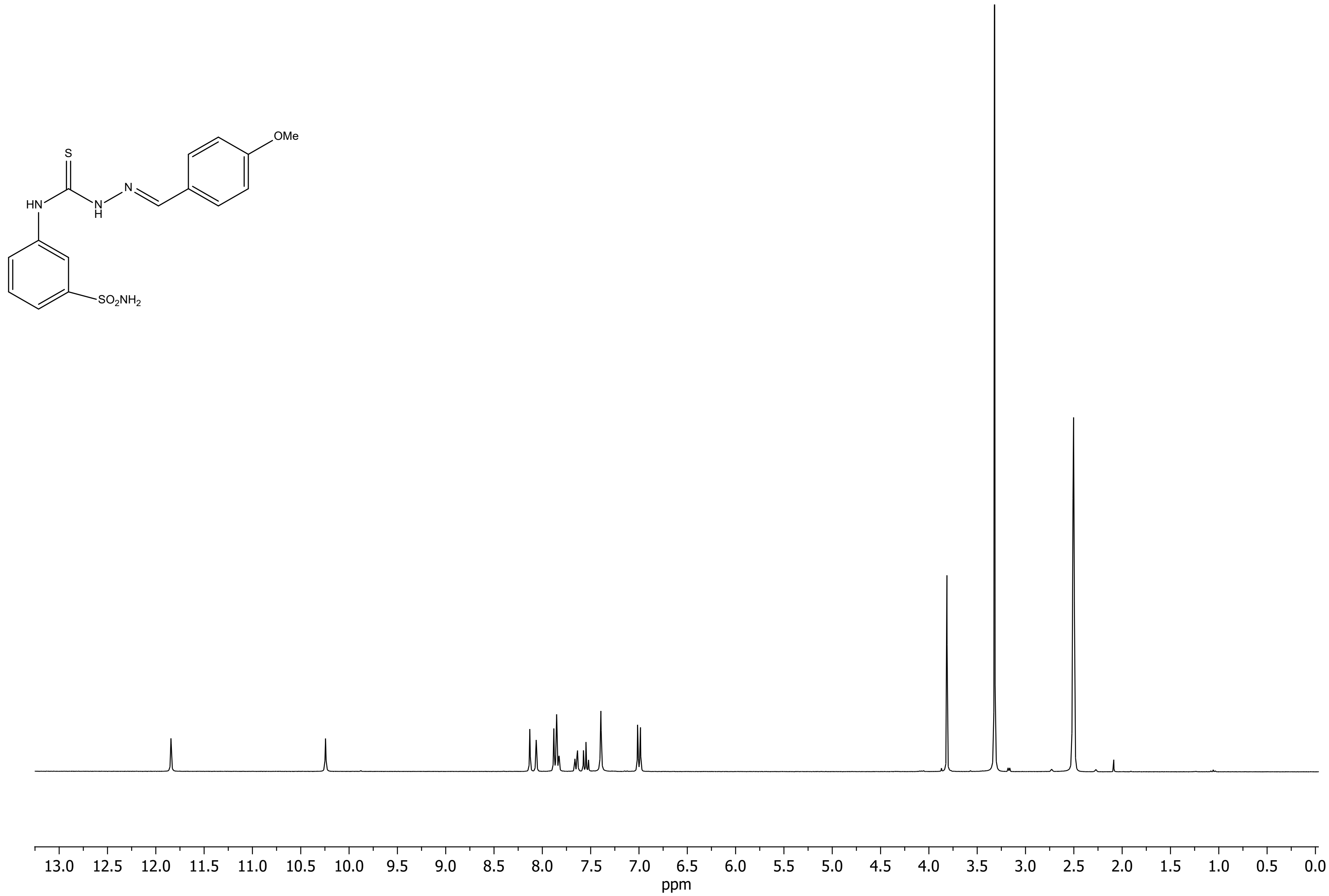

Figure S7. ^{1}H-NMR spectrum of **4h** (300 MHz, DMSO-d_6)

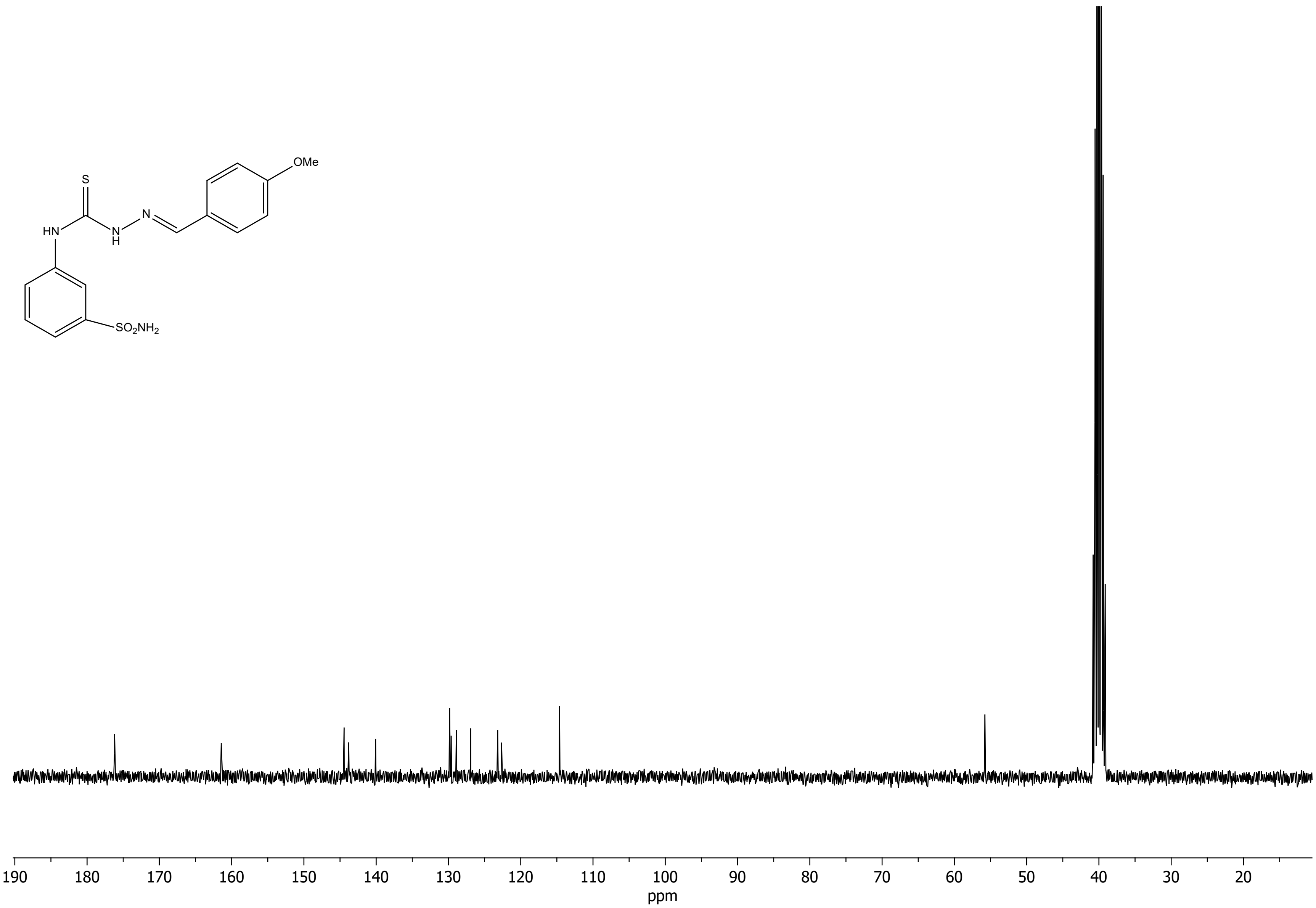

Figure S8. ^{13}C-NMR spectrum of **4h** (75.5 MHz, DMSO-d_6)

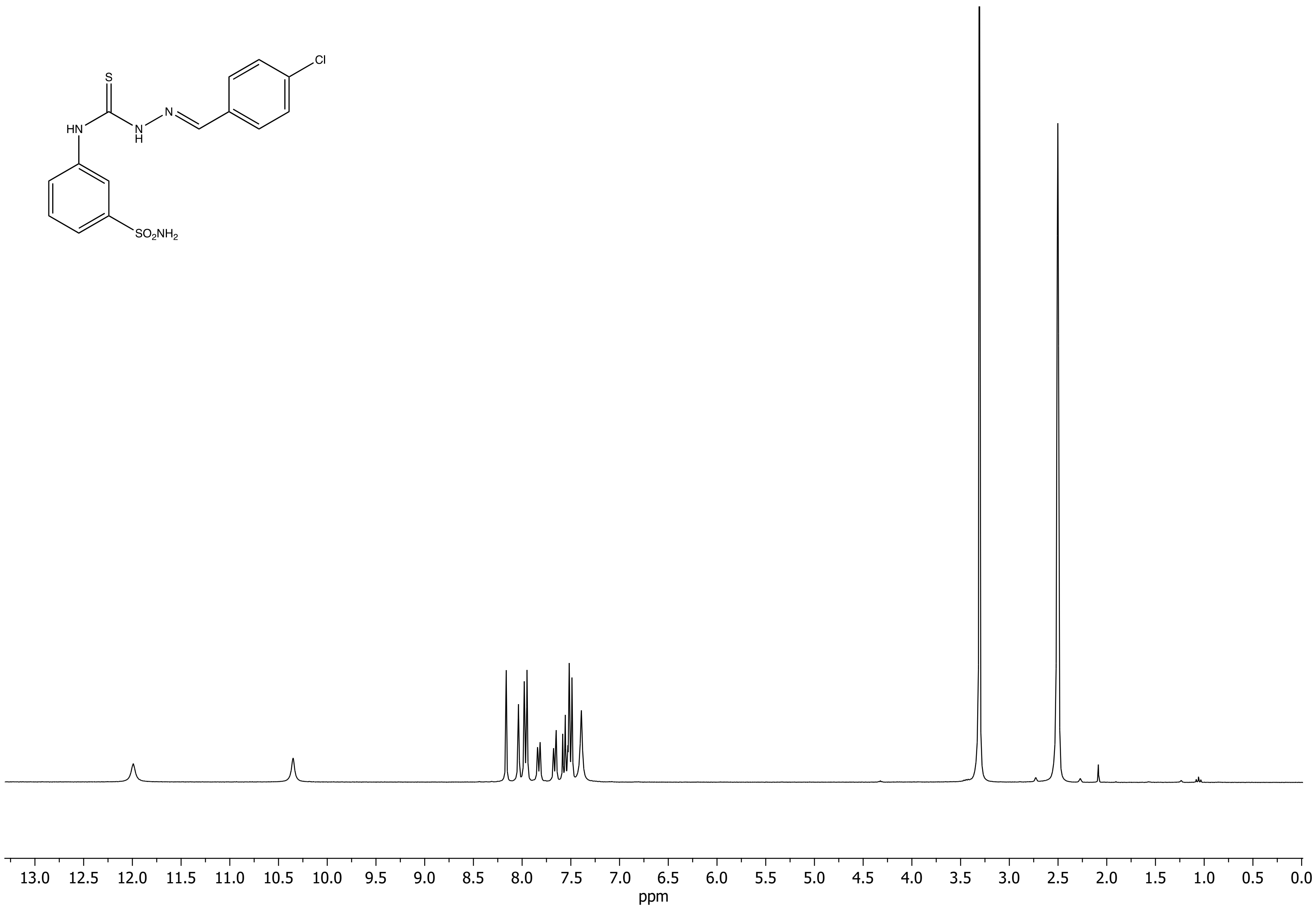

Figure S9. ^{1}H-NMR spectrum of **4j** (300 MHz, DMSO-d_6)

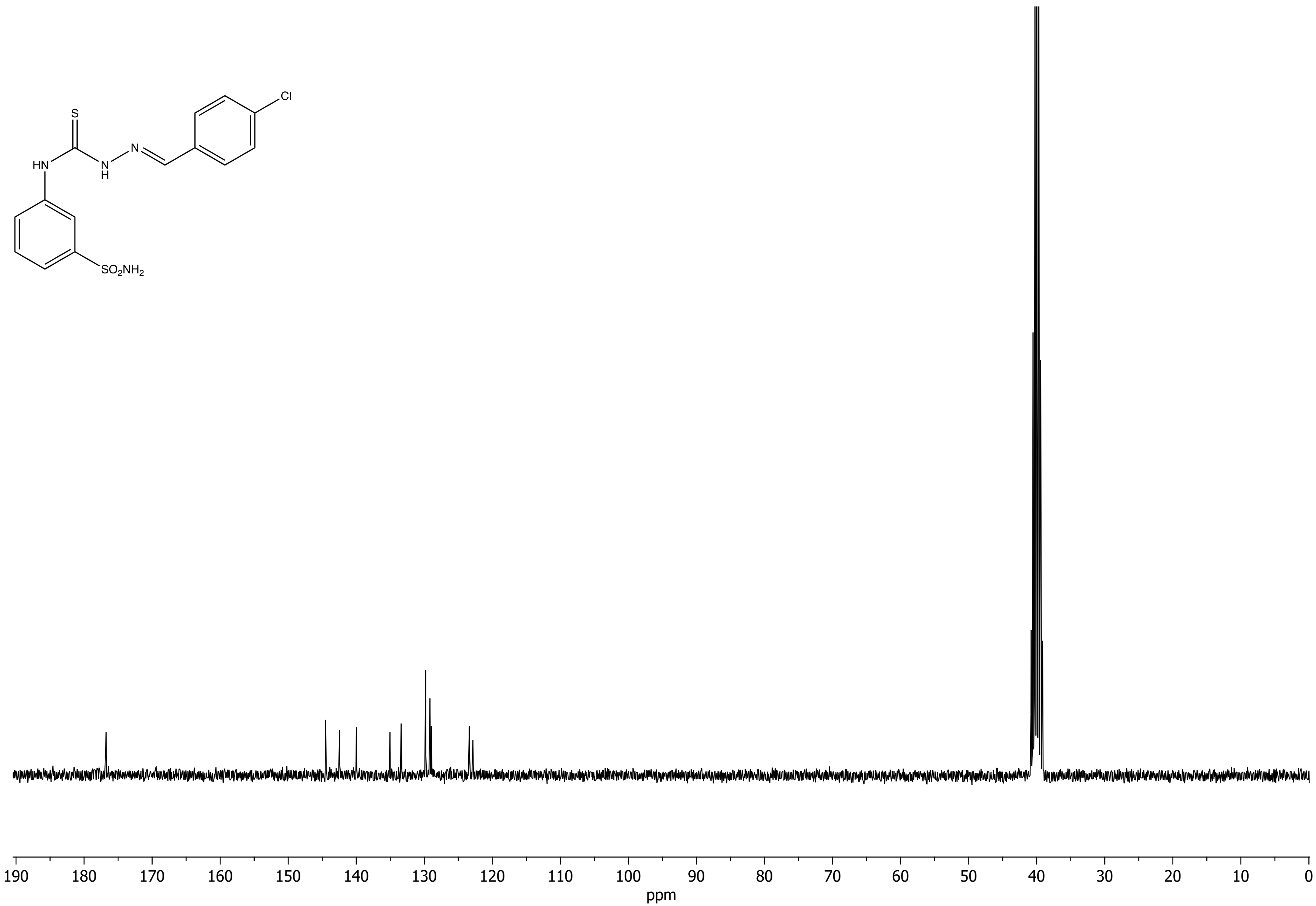

Figure S10. ^{13}C-NMR spectrum of **4j** (75.5 MHz, DMSO-d_6)

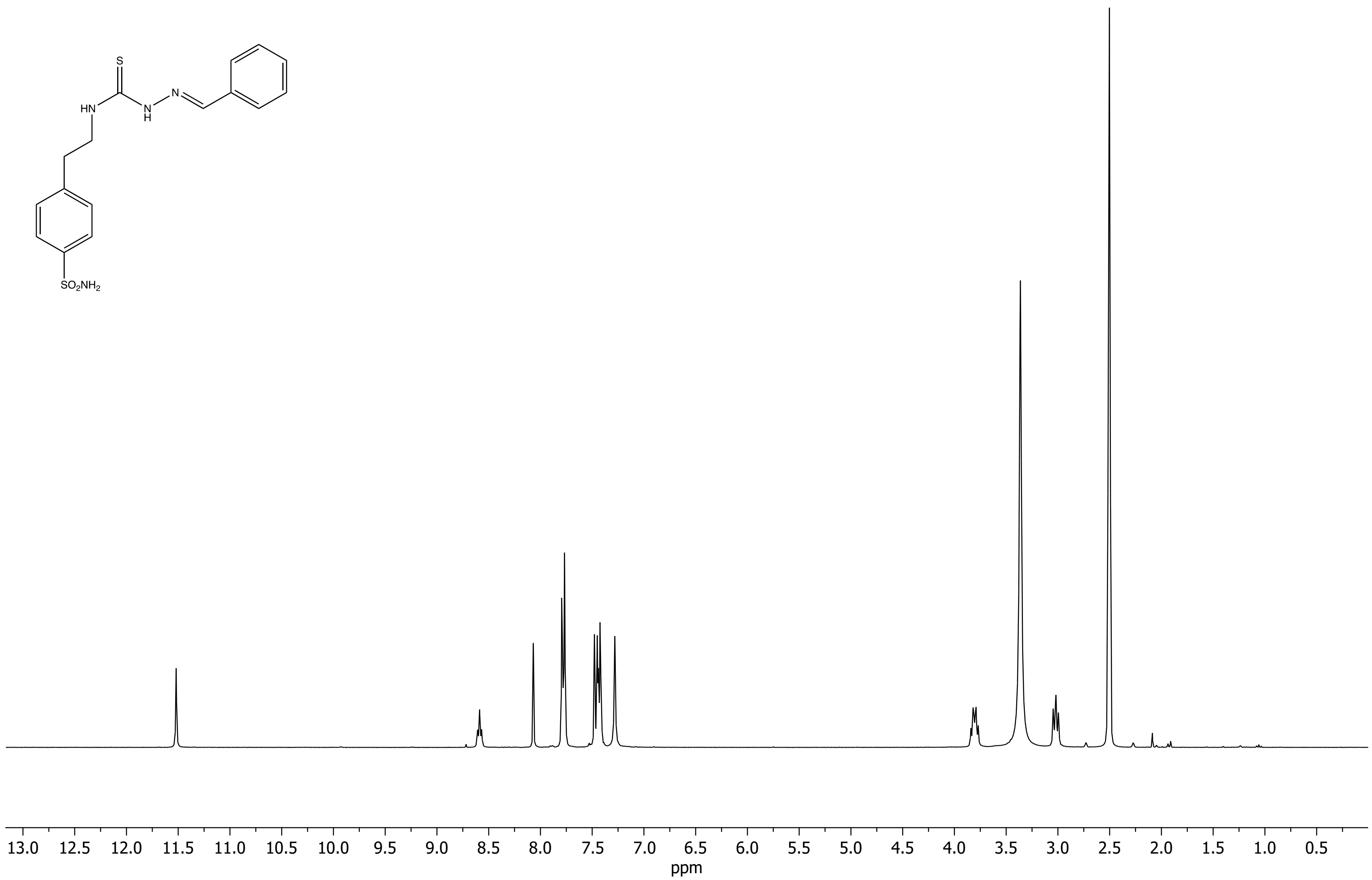

Figure S11. ^{1}H-NMR spectrum of **4l** (300 MHz, DMSO-d_6)

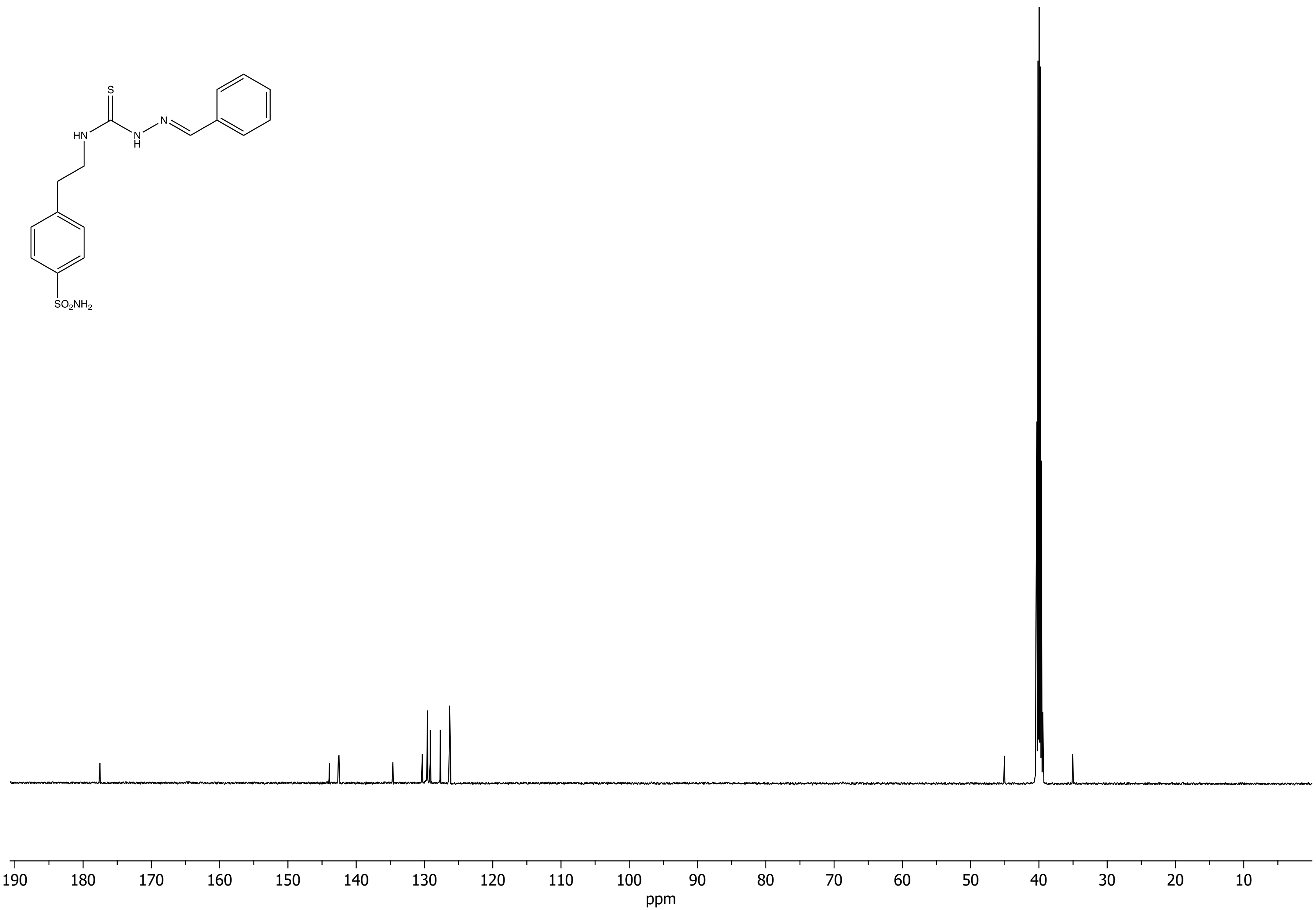

Figure S12. ^{13}C-NMR spectrum of **4l** (125.7 MHz, DMSO-d_6)

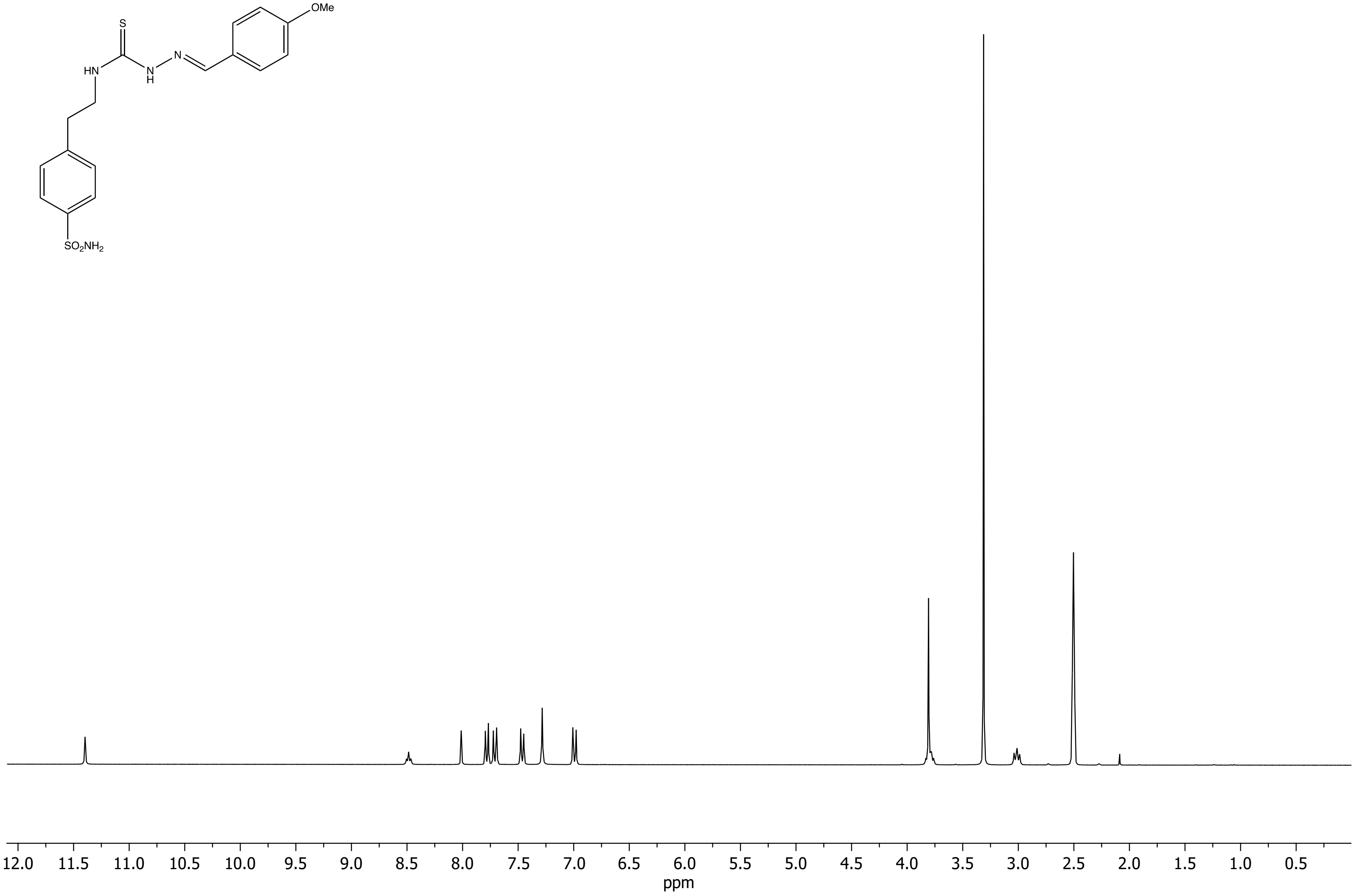

Figure S13. ^{1}H-NMR spectrum of **4m** (300 MHz, DMSO-d_6)

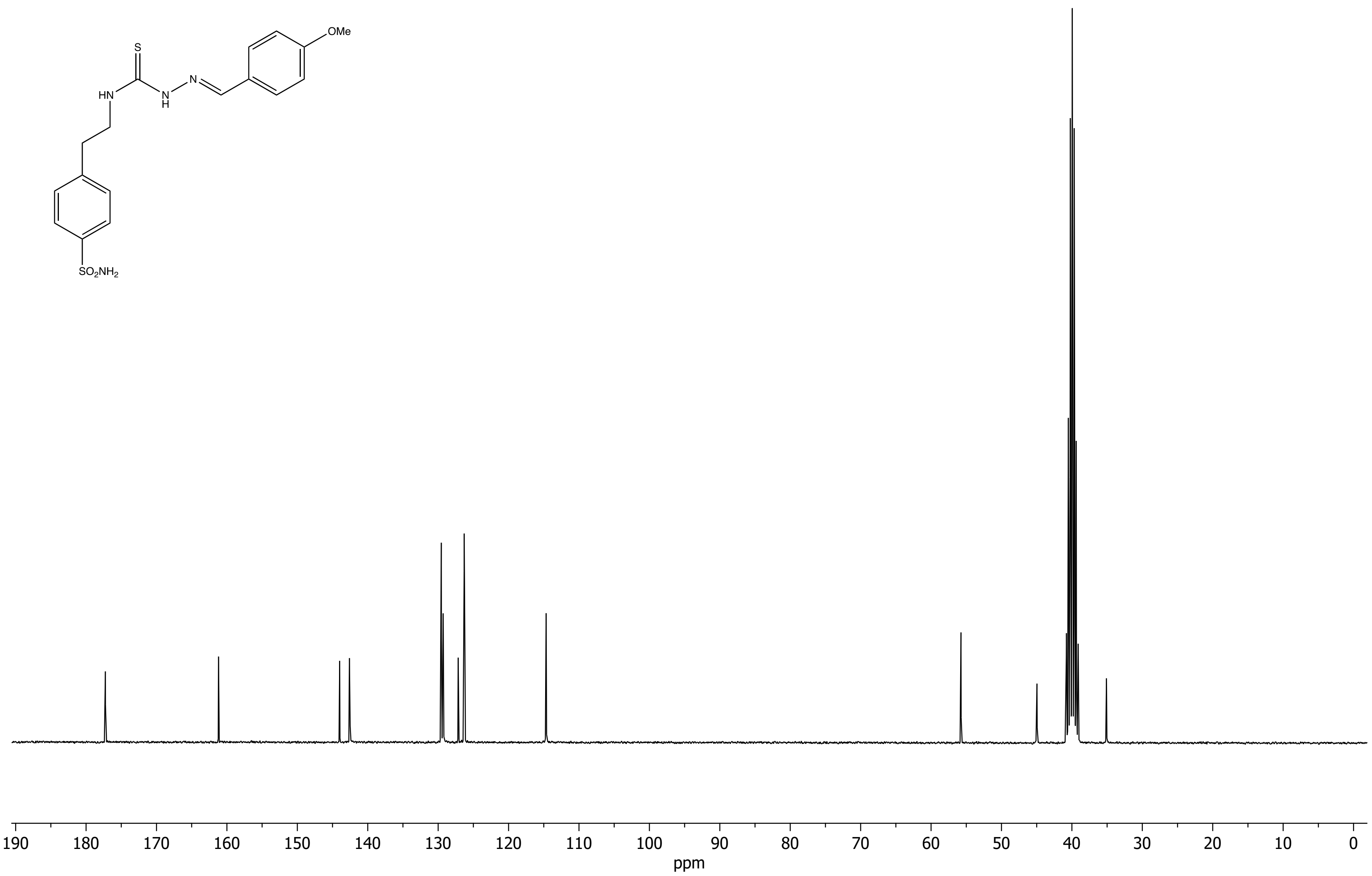

Figure S14. ^{13}C-NMR spectrum of **4m** (75.5 MHz, DMSO-d_6)

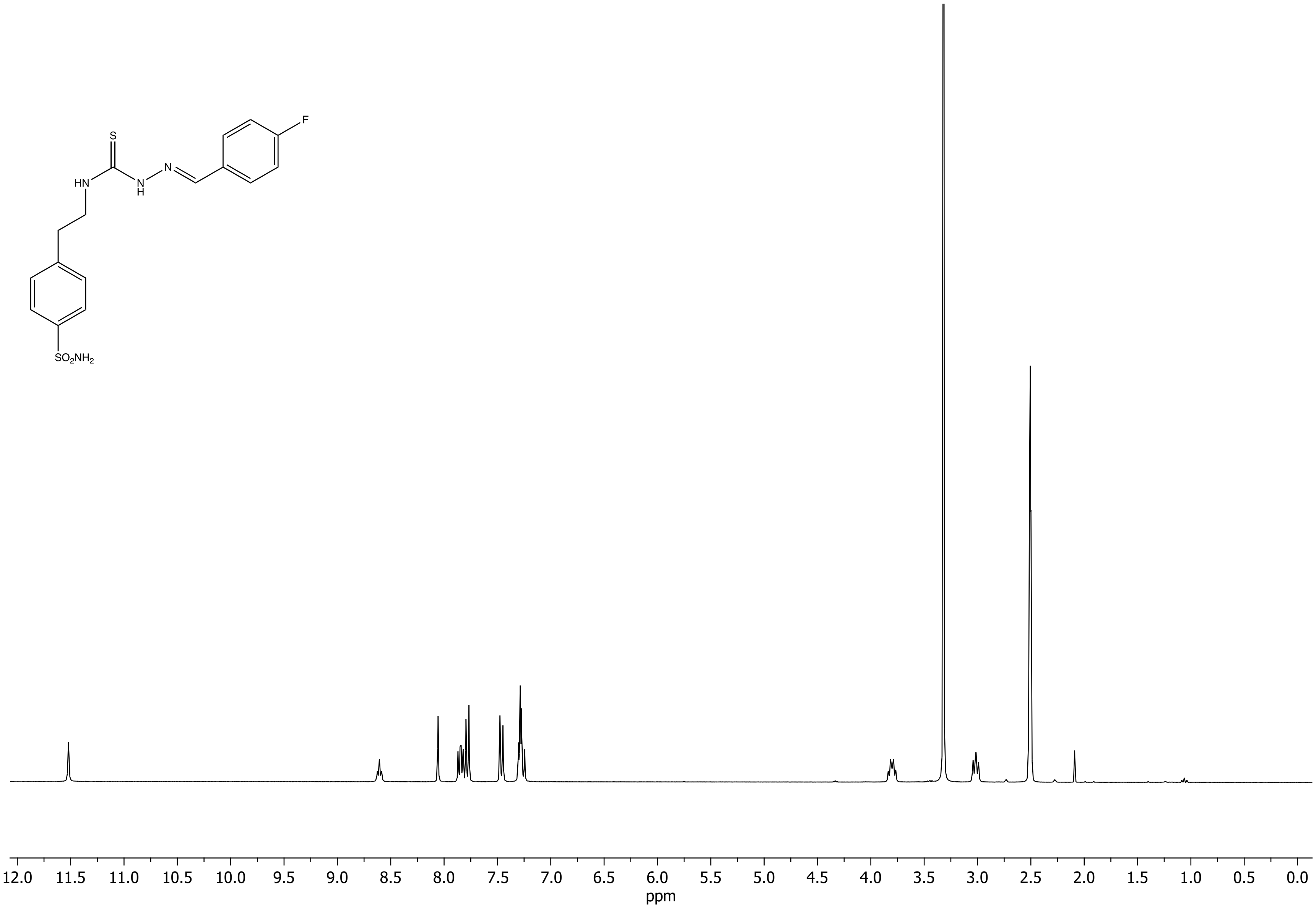

Figure S15. ^{1}H-NMR spectrum of **4n** (300 MHz, DMSO-d_6)

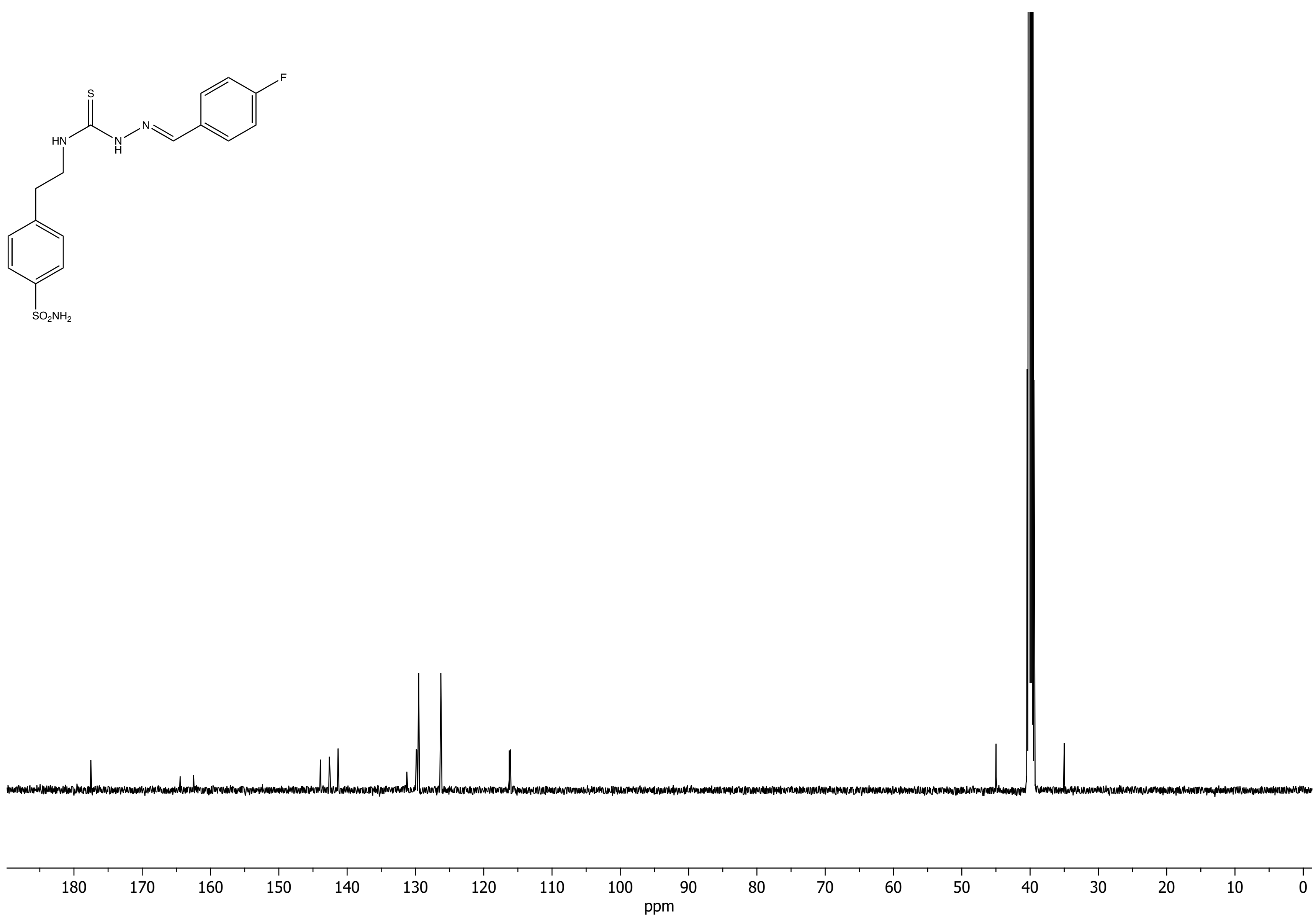

Figure S16. ^{13}C-NMR spectrum of **4n** (125.7 MHz, DMSO-d_6)

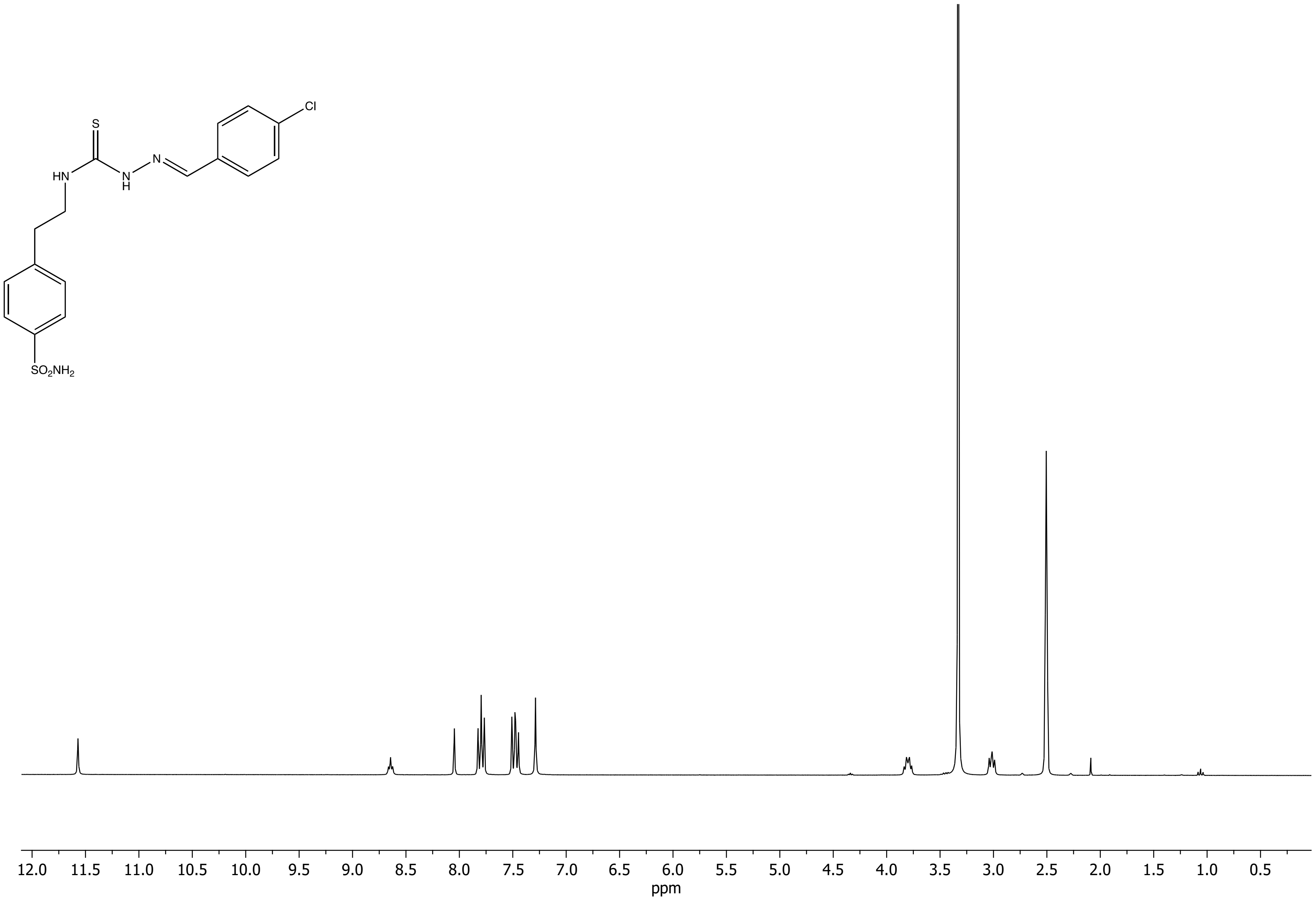

Figure S17. ^{1}H-NMR spectrum of **4o** (300 MHz, DMSO-d_6)

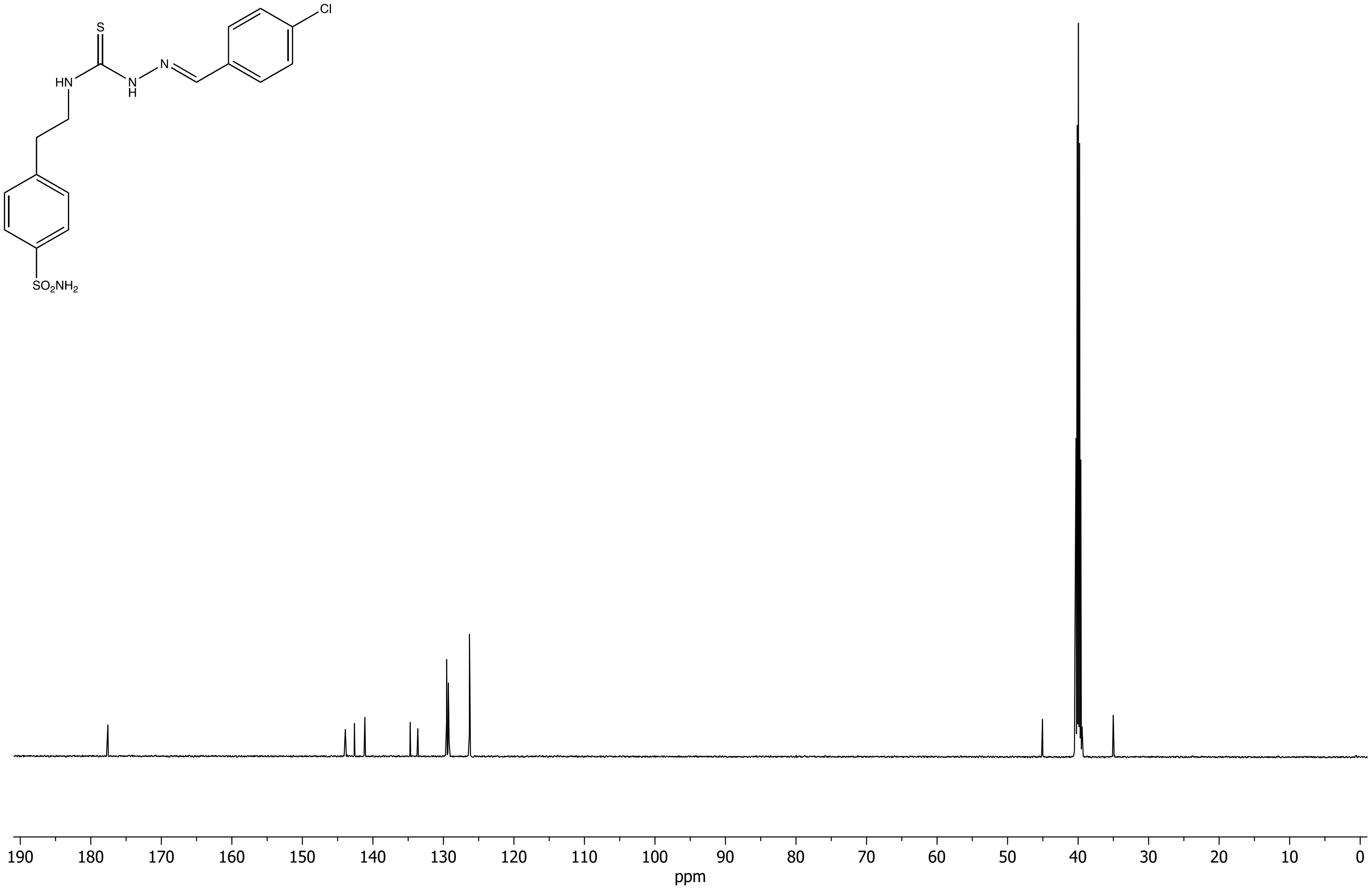

Figure S18. ^{13}C-NMR spectrum of **4o** (125.7 MHz, DMSO-d_6)

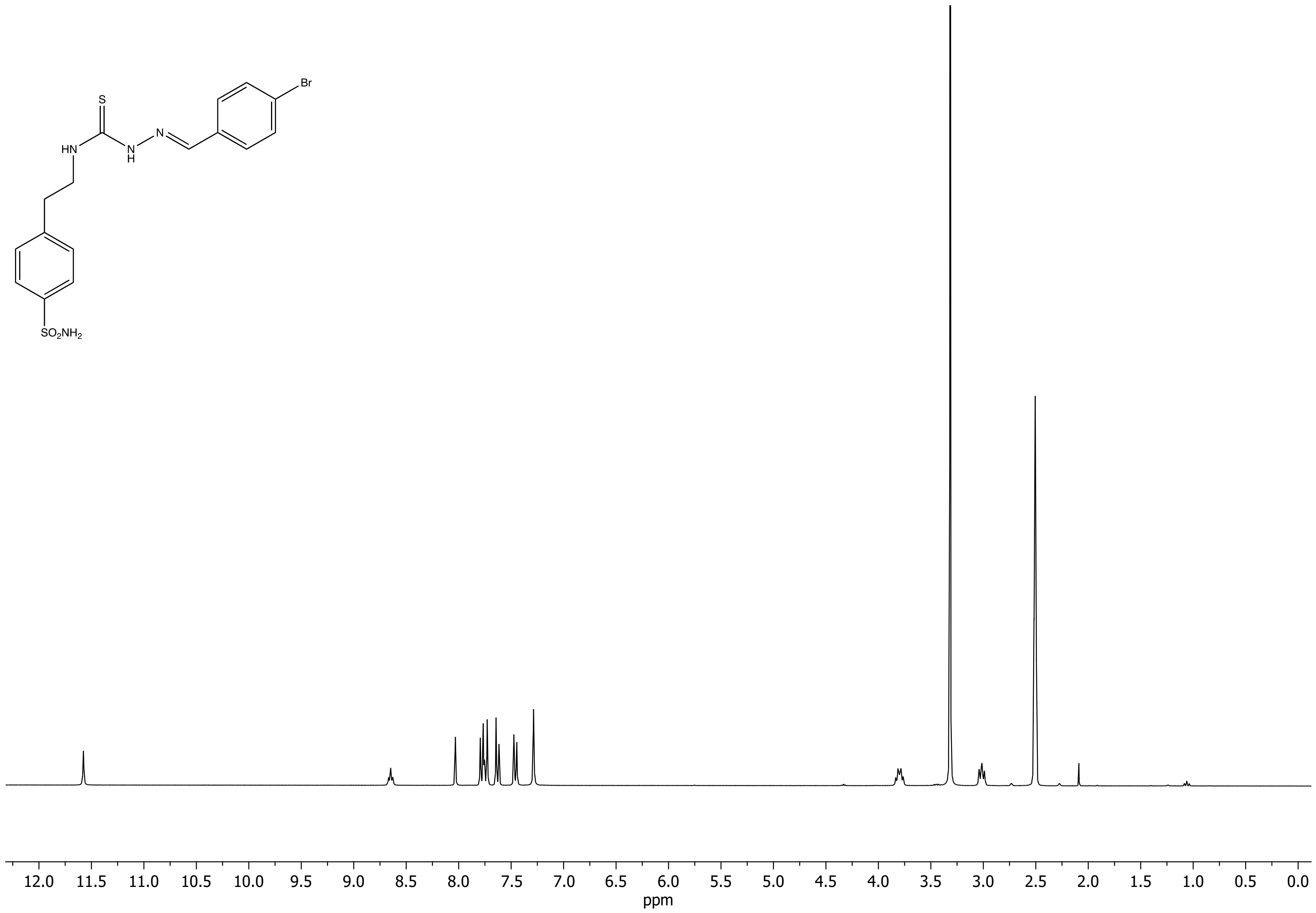

Figure S19. ^{1}H-NMR spectrum of **4p** (300 MHz, DMSO-d_6)

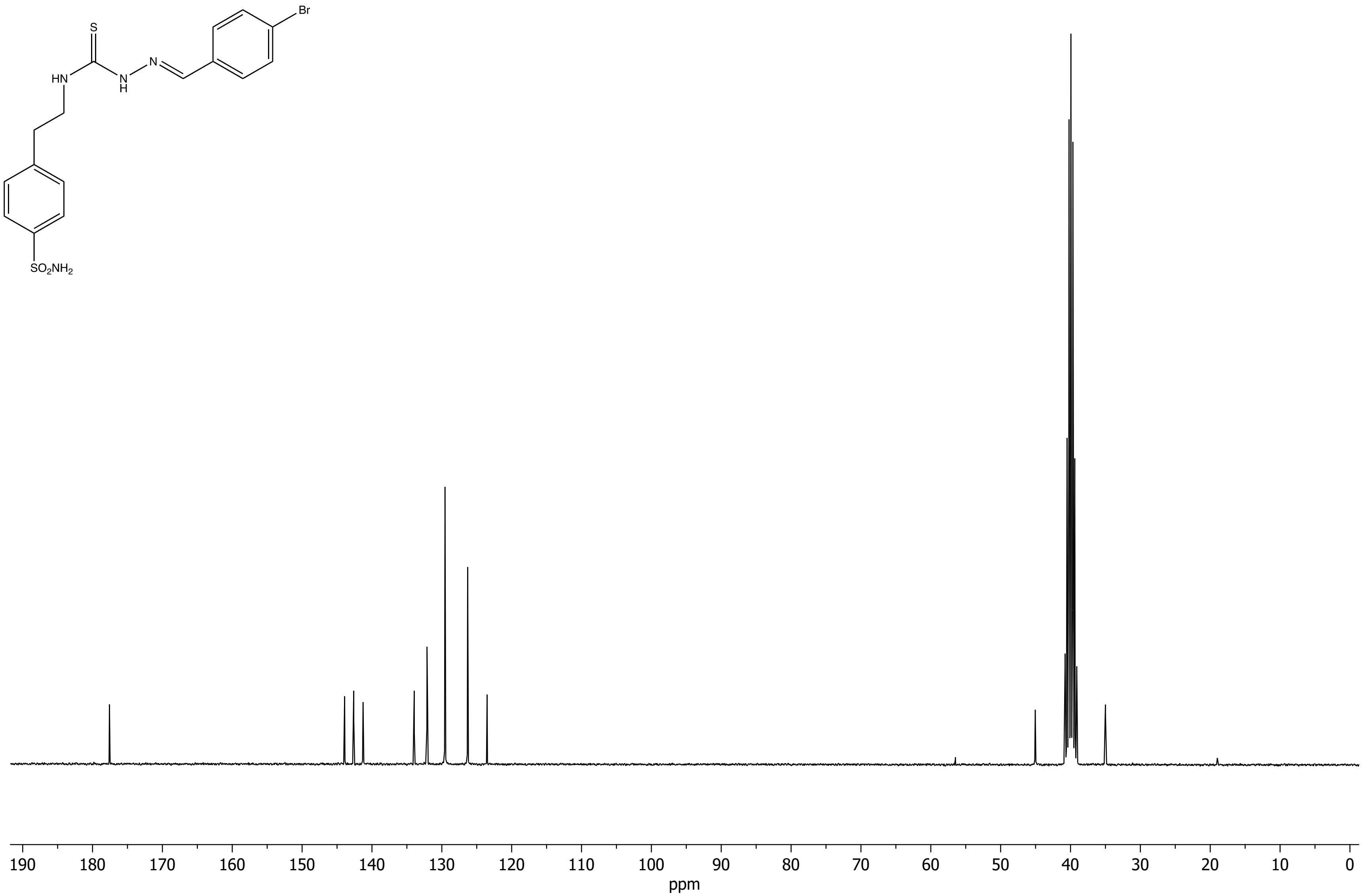

Figure S20. ^{13}C-NMR spectrum of **4p** (75.5 MHz, DMSO-d_6)

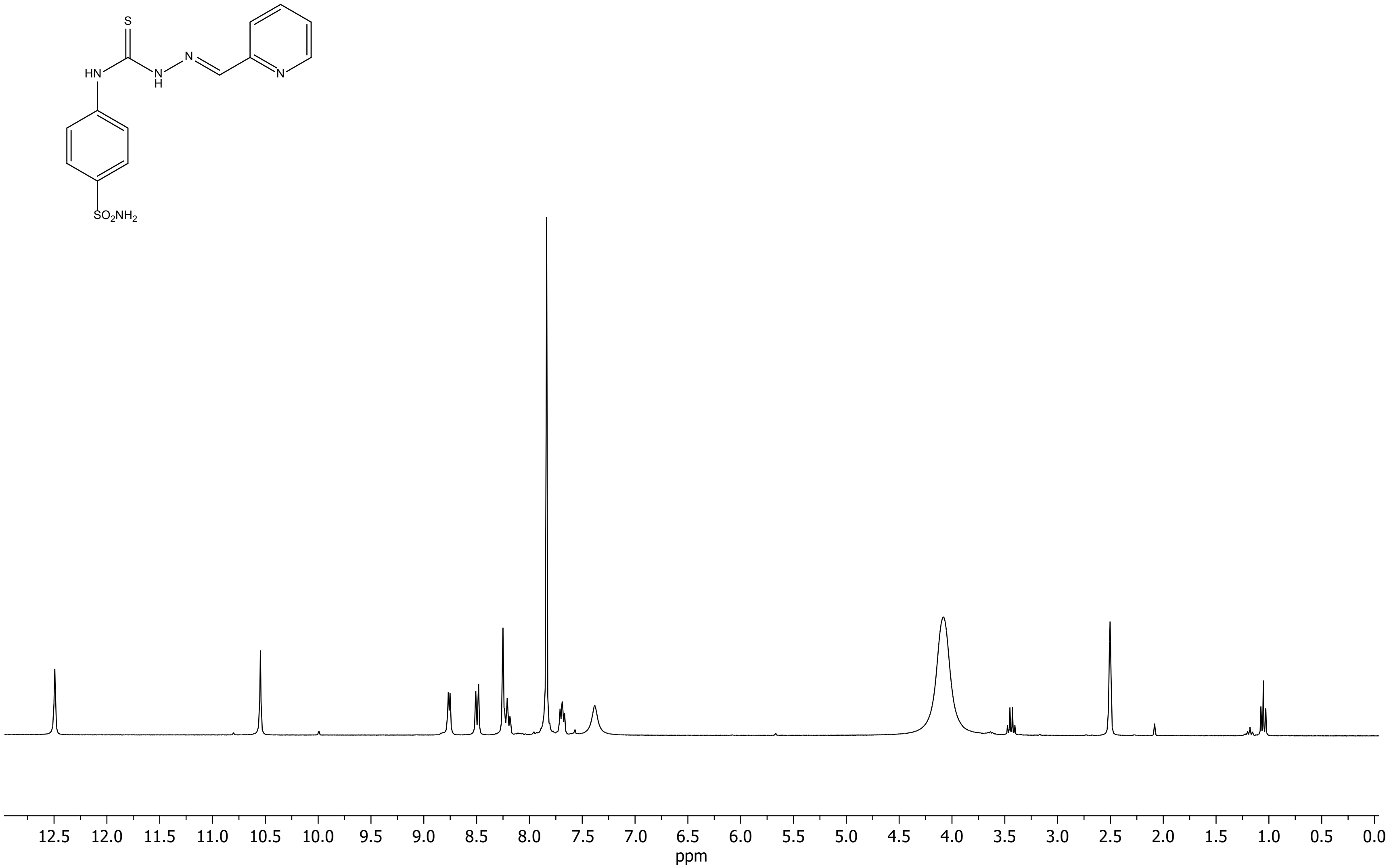

Figure S21. ^{1}H-NMR spectrum of **5a** (300 MHz, DMSO-d_6)

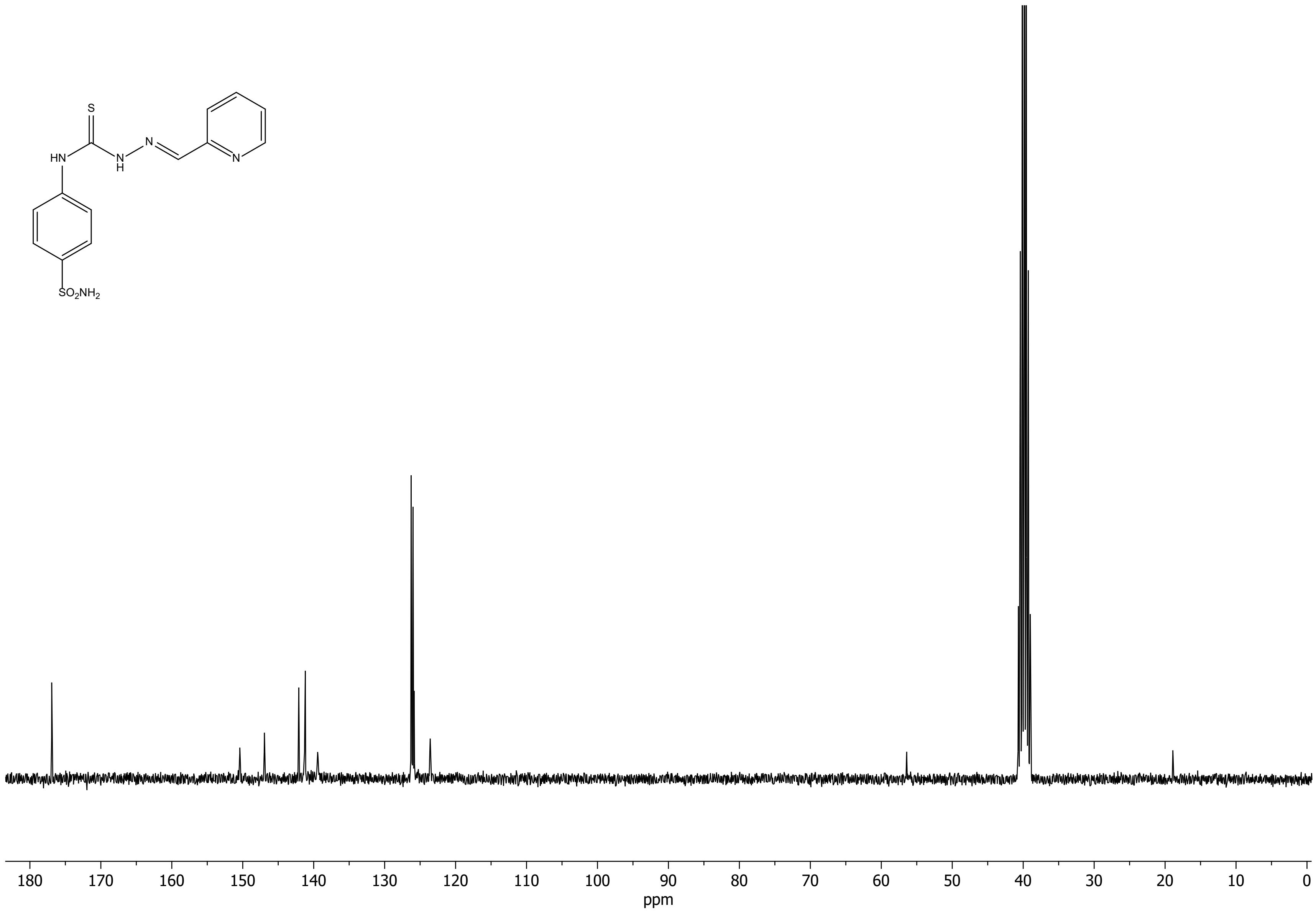

Figure S22. ^{13}C-NMR spectrum of **5a** (125.7 MHz, DMSO-d_6)

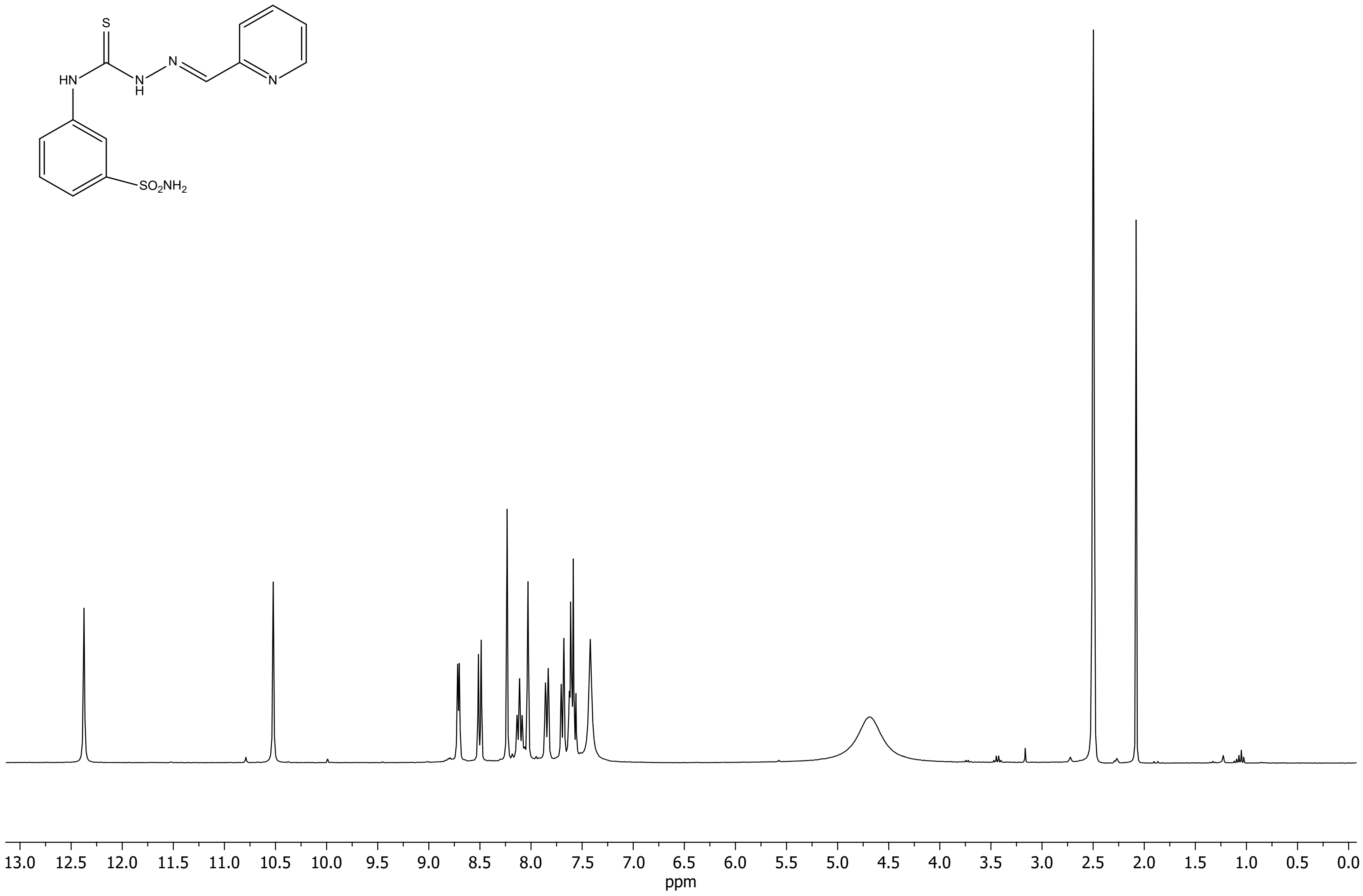

Figure S23. ^{1}H-NMR spectrum of **5b** (300 MHz, DMSO-d_6)

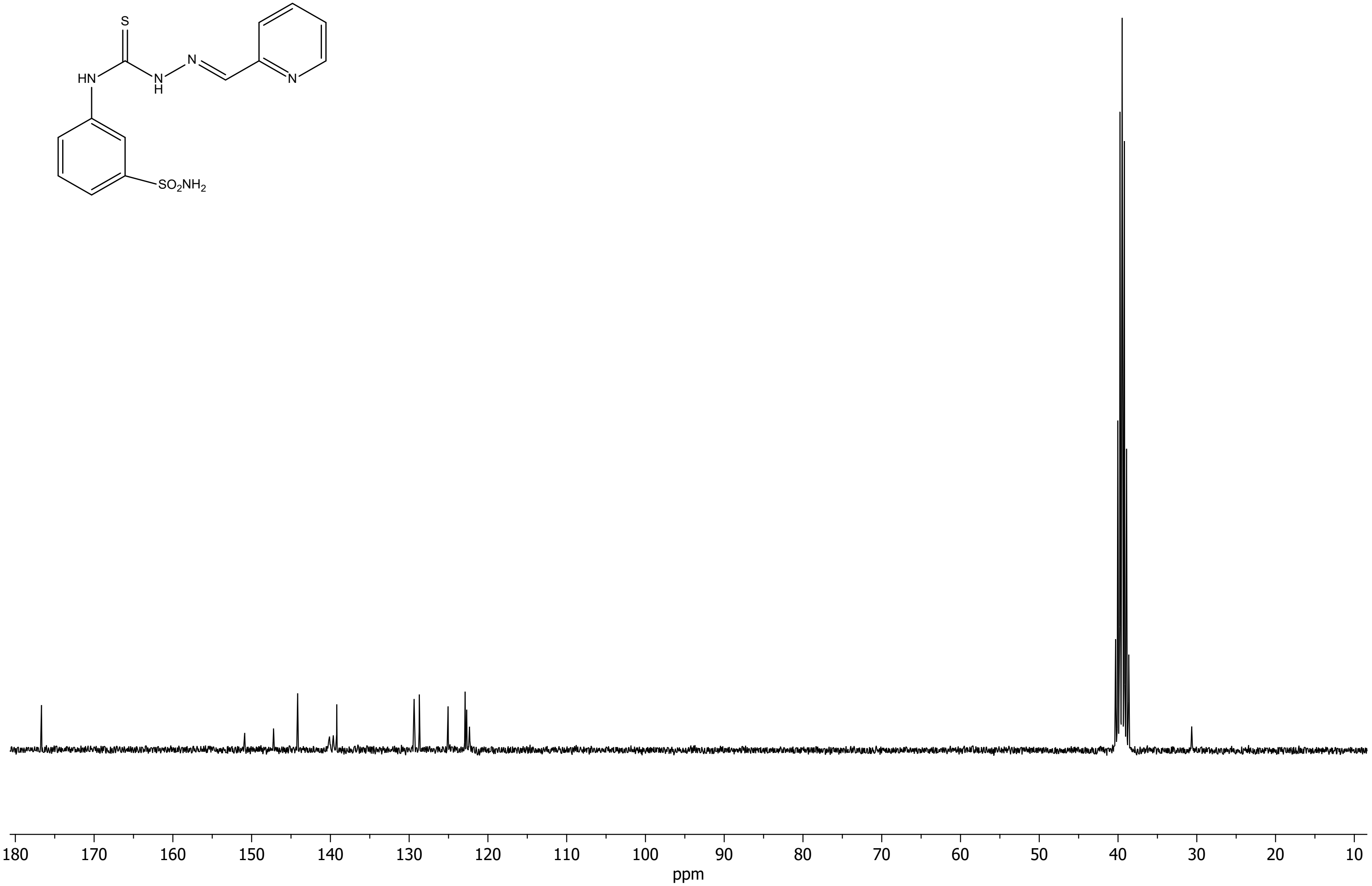

Figure S24. ^{13}C-NMR spectrum of **5b** (75.5 MHz, DMSO-d_6)

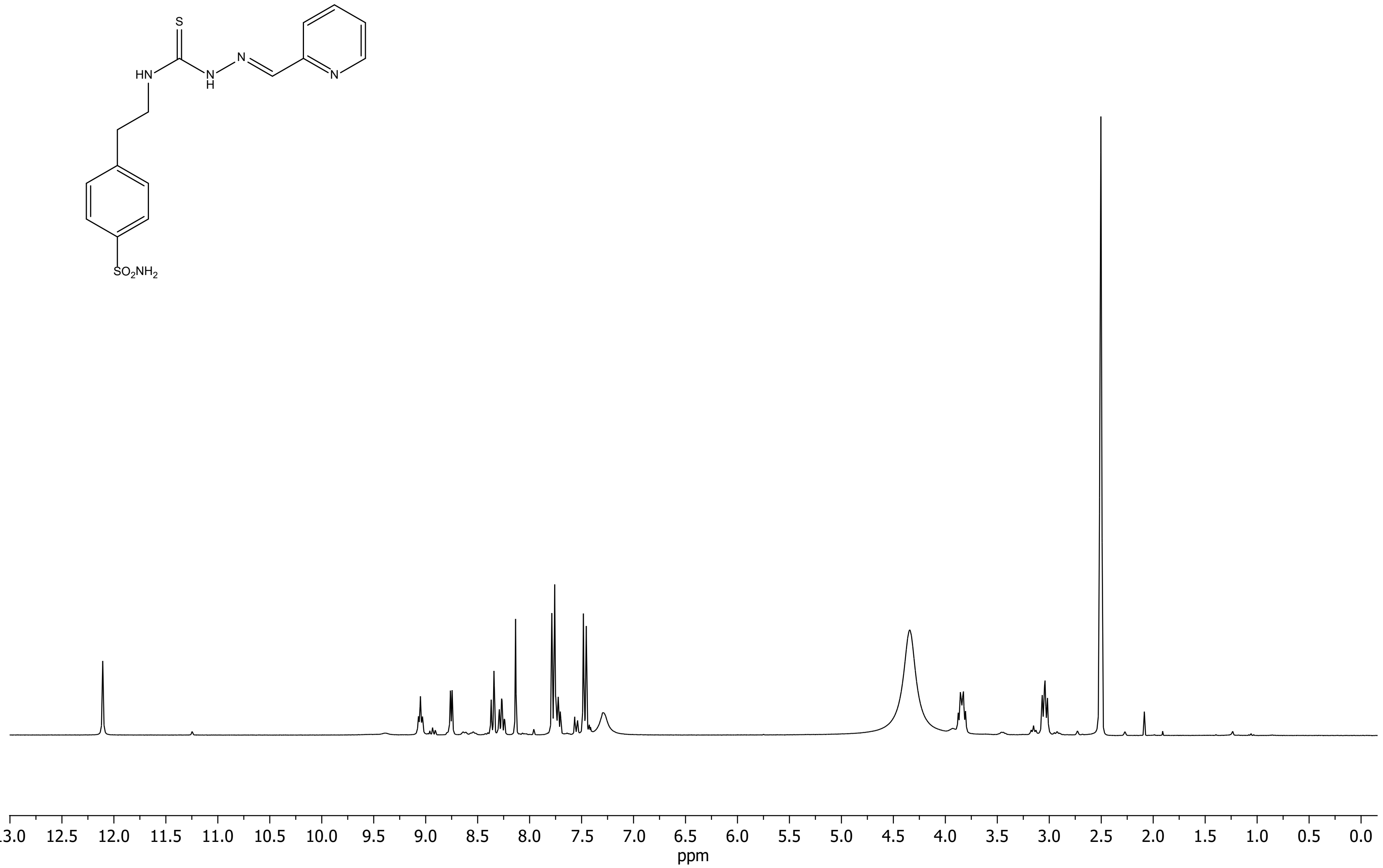

Figure S25. ^{1}H-NMR spectrum of **5c** (300 MHz, DMSO-d_6)

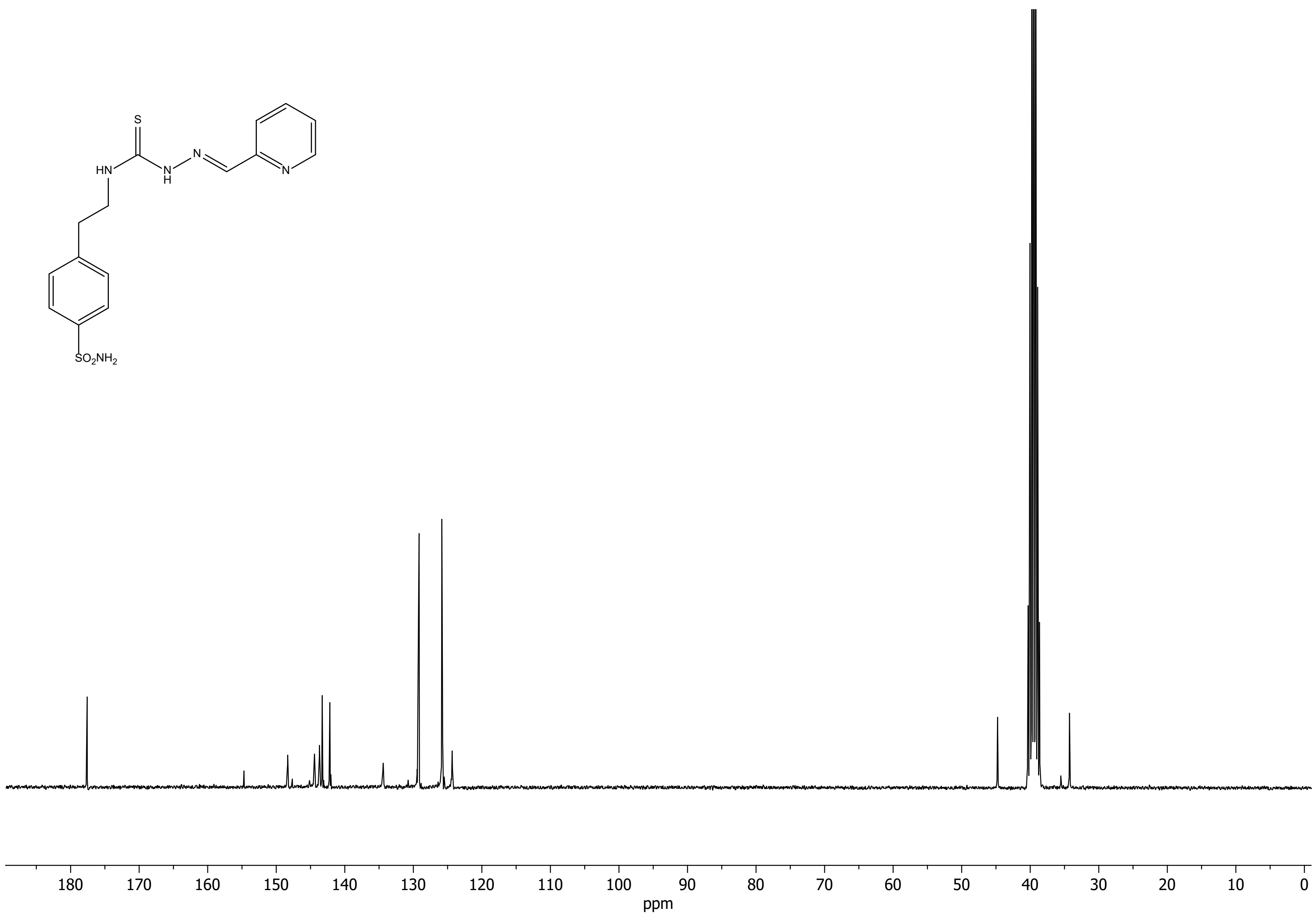

Figure S26. ^{13}C-NMR spectrum of **5c** (75.5 MHz, DMSO-d_6)
